# *Fumaria officinalis* Dust as a Source of Bioactives for Potential Dermal Application: Optimization of Extraction Procedures, Phytochemical Profiling, and Effects Related to Skin Health Benefits

**DOI:** 10.3390/plants14030352

**Published:** 2025-01-24

**Authors:** Rabiea Ashowen Ahmoda, Andrea Pirković, Violeta Milutinović, Milena Milošević, Aleksandar Marinković, Aleksandra A. Jovanović

**Affiliations:** 1 Faculty of Technology and Metallurgy, University of Belgrade, Karnegijeva 4, 11000 Belgrade, Serbia; 20214039@estudent.tmf.bg.ac.rs (R.A.A.); marinko@tmf.bg.ac.rs (A.M.); 2 Institute for the Application of Nuclear Energy INEP, University of Belgrade, Banatska 31b, Zemun, 11080 Belgrade, Serbia; andrea.pirkovic@inep.co.rs; 3 Faculty of Pharmacy, University of Belgrade, Vojvode Stepe 450, 11000 Belgrade, Serbia; violeta.milutinovic@pharmacy.bg.ac.rs; 4 Institute of Chemistry, Technology and Metallurgy, National Institute of the Republic of Serbia, University of Belgrade, Njegoševa 12, 11000 Belgrade, Serbia; milena.milosevic@ihtm.bg.ac.rs

**Keywords:** alkaloids, bioactives, extraction, *Fumaria officinalis*, optimization, plant dust, polyphenols

## Abstract

*Fumaria officinalis* (fumitory), in the form of dust, was employed as a source of bioactive extracts whose chemical profile and biological potential were investigated. According to the results of the optimization of the extraction protocol, the extract with the highest polyphenol yield was prepared using fumitory dust under the optimal conditions determined using the statistical tool, 2^3^ full factorial design: 50% ethanol and a 30:1 mL/g ratio during 120 s of microwave extraction (22.56 mg gallic acid equivalent/g of plant material). LC-MS and spectrophotometric/gravimetric analyses quantified the polyphenol, flavonoid, tannin, alkaloid, and protein contents. Caffeoylmalic acid, quercetin dihexoside, quercetin pentoside hexoside, rutin, and methylquercetin dihexoside were the most dominant compounds. The highest total flavonoid, condensed tannin, alkaloid, and protein yields were determined in the extract prepared using microwaves. In addition to the proven antioxidant potential, in the present study, the anti-inflammatory activity of fumitory extracts is also proven in the keratinocyte model, as well as a significant reduction of H_2_O_2_-induced reactive oxygen species production in cells and the absence of keratinocyte cytotoxicity. Thus, detailed chemical profiles and investigated biological effects related to skin health benefits encourage the potential application of fumitory dust extracts in dermo-cosmetic and pharmaceutical preparations for dermatological circumstances.

## 1. Introduction

*Fumaria officinalis* L. (common fumitory or earth smoke, Fumariaceae) is a scrambling annual plant, disturbed and cultivated throughout Europe. It has smooth, slender, and branched stems of variable heights (10–100 cm), containing bare gray–green, feathery, and alternate leaves, and lateral and terminal racemes of purplish pink, as well as a smoky appearance of the whole plant. The plant is a component of various phytotherapeutic formulations in the European ethnobotany used in hepatobiliary dysfunction, illnesses of gastrointestinal and urogenital tracts, cancer, rheumatism, high blood pressure, and skin disorders [1,2,3,4,5]. According to Duke et al. [6], indications for the use of fumaric acid-enriched plants such as fumitory in traditional medicine for skin and scalp disorders include acne, alopecia, eczema, infection, inflammation, itch, leprosy, psoriasis, and scabies. *F. officinalis* has been empirically used to treat inflammatory skin disorders since the 17th century [7]. Its aerial flowering parts have traditionally been used for hepatic and gallbladder diseases and as a cholagogue, antihypertensive, spasmolytic, respiratory stimulant, and anti-arteriosclerosis agent as well. In addition, the plant has been employed for constipation, liver detoxification, to promote urinary and digestive elimination functions, symptomatic relief of digestive disorders such as dyspepsia and flatulence, dyskinesia of the biliary duct, pain in case of cholelithiasis when surgery is not possible, pain in case of cholecystitis and cholangitis, post-cholecystectomy syndrome and post-hepatic syndrome with cholestasis [1,4,8,9]. Several studies have shown the antimicrobial, antioxidant, antispasmodic, laxative, anthelmintic, anticoagulant, cholagogue, cytotoxic, antidiabetic, analgesic, neural, and sedative effects of *F. officinalis* [1,2,10,11,12,13,14]. The plant was traditionally used to remove skin blemishes and treat skin diseases, including chronic eczema, cutaneous eruptions, psoriasis, scabies, and other dermatological issues, due to the presence of alkaloids and polyphenols [5,9,12,15]. According to the European Medicines Agency (EMA) and Herbal Medicines and Products Committee (HMPC)’s monograph on herbal medicinal products, the indication for the traditional fumitory herbal medicinal product includes increased bile flow for the relief of symptoms of indigestion (such as the sensation of fullness, flatulence, and slow digestion) [16]. Low-polar fumitory extracts exhibited cytotoxic effects on two leukemia and nine multiple myeloma cell lines [2]. The pharmacological activities mentioned can be attributed to the high content of polyphenols (flavonoids, phenolic acids, and tannins) and isoquinoline alkaloids present in this species [1,2,3,9,10]. Phytochemical studies of *F*. *officinalis* have dealt with a range of bioactive compounds present in various plant extracts. Regarding phytochemical investigations, previously diverse extracts of *F. officinalis* herb were analyzed, releasing the presence of flavonoids (quercetin and its glycosides as well as derivatives), phenolic acids (chlorogenic, *p*-coumaric, ferulic, and caffeoylmalic acids), and isoquinoline alkaloids of the protopine (with protopine being the predominant alkaloid), tetrahydroprotoberine, and spyrobenzilisoquinoline types [17,18,19,20,21]. Regarding pharmacological studies, an ethanol extract of *F. officinalis* demonstrated significant effects in an *in vivo* ethanol-induced gastric ulcer model, compared to the control and esomeprazole groups [18].

Due to their potential benefits for human health, plant-based bioactive components from waste hold significant prospects for use as pharmaceutical agents, dietary supplements, functional foods, food additives, or cosmetic active ingredients. Utilizing herbal waste as raw materials for extraction can reduce production costs and environmental impact. Plant waste includes leaves, seeds, pomaces, peels, herbal dust, or other by-products of the manufacturing process. Waste is plentiful in lipids, sugars, fibers, vitamins, and polyphenols that can be implemented in different formulations [22,23]. Several recent studies successfully extracted bioactive products (essential oils and extracts) from the herbal dust of wild thyme and sage [24,25,26,27]. The herbal matrix containing particles lower than 0.5 mm represents plant dust and cannot be an integral part of tea products for the market, for trade or for sale (regulations on the quality of tea, herbal tea, and their products of the Republic of Serbia) [28]. Additionally, the particle size of plant material can significantly affect the extraction yield. Namely, the highest content of target compounds is principally extracted using the finest particles, due to the increased active surface area (the highest level of destroyed plant cells) and the enhanced contact of the herbal matrix with the extraction medium, which both consequently lead to the reduction in extraction time as well [29]. All mentioned advantages encourage the employment of herbal dust for the extraction of biologically active compounds. Bioactive components of herbal dust can be extracted by employing conventional techniques, such as Soxhlet extraction (for lipid extraction), heated reflux extraction (to enhance the extraction efficiency of thermostable compounds), percolation (for the matrix that requires a higher amount of the extraction solvent), or maceration (for the extraction of thermosensitive compounds). Despite their simple operation and cost-effectiveness, conventional extraction procedures have significant limitations: the requirement for an extended extraction duration, the use of large volumes of potentially hazardous organic solvents, and a longer extraction time [22,25,29]. To enhance the yield of extractable bioactives and shorten the extraction time, modern approaches are commonly employed (ultrasound-, microwave-, infrared-, and enzyme-assisted extractions, as well as pulsed electric field, pressurized liquid, supercritical fluid, and subcritical fluid extractions). Nevertheless, the novel techniques mentioned also possess disadvantages, including high equipment costs and energy consumption, lower selectivity (microwave extraction), sensitivity to changes in pH or temperature (enzyme extraction), and accelerated hydrolysis of the compounds (subcritical fluid extraction) [22,29].

Based on the literature search, the medicinal applications of *F. officinalis* in dermatological conditions and infections have not been adequately supported by research. Thus, it is necessary to investigate fumitory more in terms of various biological activities and bioactive phytochemical compounds. The present research aimed to optimize the extraction of bioactive principles from *F. officinalis* dust via varying factors of interest (ethanol content in extraction medium, solvent-to-solid ratio, and extraction time), by performing traditional and novel extraction procedures (maceration and heat-, ultrasound-, and microwave-assisted extractions, i.e., M, HAE, UAE, and MAE, respectively), and to investigate the phytochemical profile and biological potential of the obtained extracts. The optimal extraction conditions for achieving the highest polyphenol yield were obtained by employing a full factorial design as a statistical tool. Qualitative and quantitative analyses of individual compounds of *F. officinalis* extracts were performed using the LC-MS method as well. The quantification of total flavonoid, condensed tannin, alkaloid, and protein yields and the plant’s antioxidant, anti-inflammatory, and keratinocyte viability effects, due to the above-mentioned traditional use of the plant in skin disorders [5,6,7], were the focus of the present study. Therefore, the study can provide data on the optimal extraction conditions and method for obtaining potent extracts from fumitory dust, and evidence of their chemical profile and biological potential, to be used in the pharmaceutical and dermo-cosmetic formulations for skin disorders.

## 2. Results

The influence of different ethanol contents in water (0, 50, and 70%), solvent volumes (solvent-to-solid ratio of 20:1, 30:1, and 40:1 mL/g), and extraction times (30, 60, and 90 min of M, 15, 30, and 45 min of HAE, 5, 15, and 30 min of UAE, and 60, 120, and 180 s of MAE) on the polyphenolic yield in fumitory extracts was examined employing one-way ANOVA analysis, followed by Duncan’s post hoc test. Following the results obtained using the mentioned statistical tools, two levels of each tested parameter (ethanol content, solvent volume, and time) were chosen for future experimental design, i.e., 2^3^ full factorial design (three parameters at two promising levels), to determine the optimal conditions for reaching the highest TPC separately for each employed extraction technique. After that, four selected fumitory extracts were prepared using the optimal extraction conditions (determined in the 2^3^ full factorial design) for M, HAE, UAE, and MAE. The selected extracts were further examined in terms of chemical characterization (determination of total flavonoid, tannin, alkaloid, and protein contents and LC-MS analysis), antioxidant and anti-inflammatory capacities, and potential cytotoxicity on the HaCaT cells.

### 2.1. Preliminary Screening of Extraction Parameters

**Figure 1:**
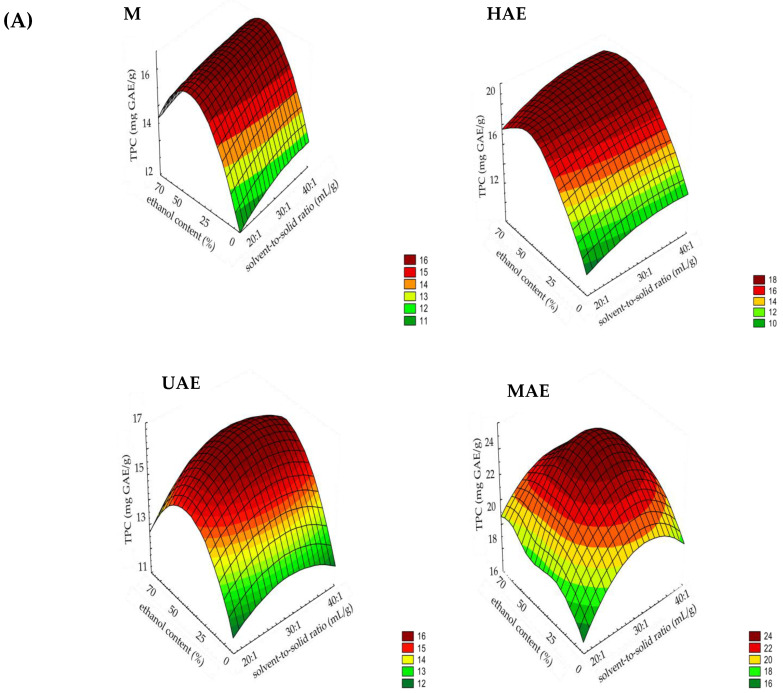

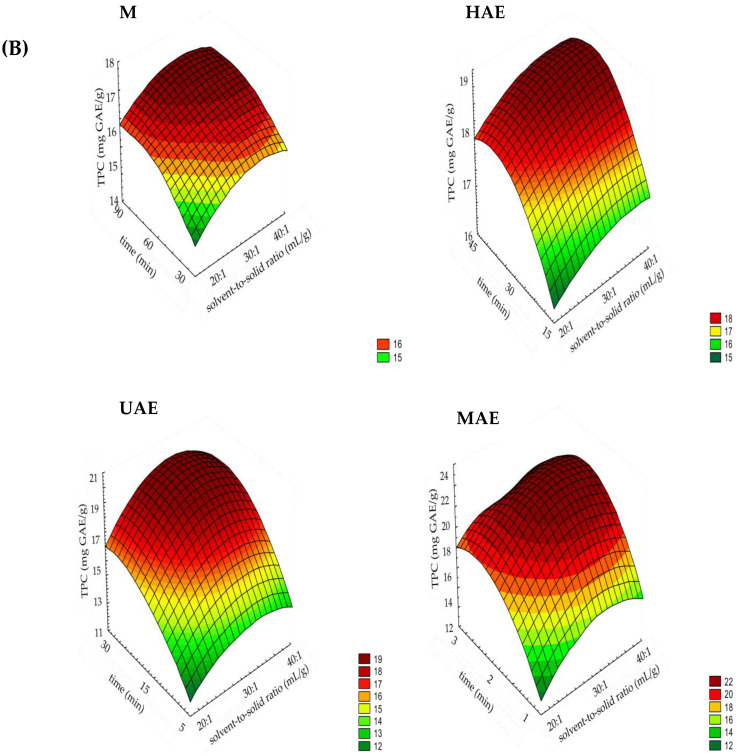

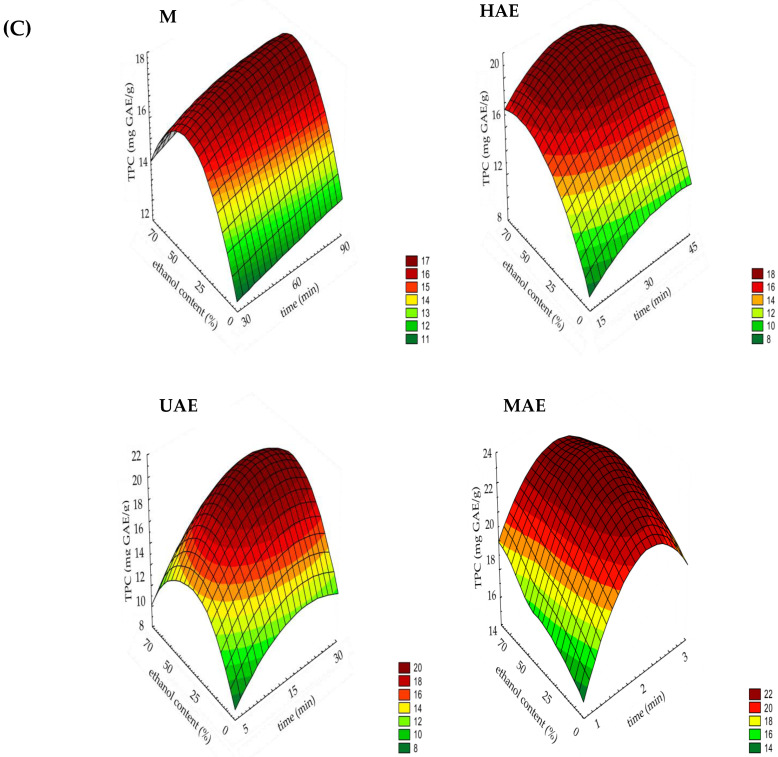
Surface plots of ethanol content, solvent-to-solid ratio, and extraction time’s impact on the total polyphenol content (TPC) of *Fumaria officinalis* extracts obtained in maceration (M) and heat-, ultrasound-, and microwave-assisted extractions (HAE, UAE, and MAE, respectively). (**A**) Ethanol content vs. solvent-to-solid ratio, (**B**) time vs. solvent-to-solid ratio, and (**C**) ethanol content vs. time; GAE, gallic acid equivalent.

The extraction of target compounds can be significantly affected by the extraction factors and their employed levels; therefore, preliminary screening of parameters—ethanol content in the extraction medium, solvent-to-solid ratio, and extraction times—was carried out in the statistical software STATISTICA 7.0., using tools one-way ANOVA and Duncan’s post hoc test. The surface plots of ethanol content, solvent-to-solid ratio, and time impact on the TPC of all prepared *F. officinalis* extracts in M, HAE, UAE, and MAE are shown in Figure 1. Additionally, the influence of ethanol content in the extraction solvent, solvent-to-solid ratio, and time on the polyphenol yield of fumitory extracts expressed via statistical significance is shown in Figure S1.

In M and UAE, the addition of ethanol in a water medium caused an increase in the polyphenol yield of up to 50% of ethanol, while in the case of 70% ethanol extracts, the TPC was lower in comparison to 50% ethanol parallels, but higher than in water extracts (Figure 1A,C). The same effect was observed in the results of one-way ANOVA and Duncan’s post hoc test (Figure S1), where different ethanol contents significantly affected the TPC at all levels. The trend was as follows: 50% ethanol > 70% ethanol > water. In HAE and MAE, the water–ethanol mixture also provided a higher polyphenol concentration compared to water (Figure 1A,C); however, there was no difference between 50 and 70% ethanolic extracts confirmed by one-way ANOVA or Duncan’s post hoc test (Figure S1). Hence, 50 and 70% fumitory ethanol extracts showed the highest polyphenol yield in all employed extraction techniques, and they were included in future 2^3^ experimental design.

As can be seen from Figure 1A,B, in M, an increase in the solvent-to-solid ratio from 20:1 to 30:1 mL/g resulted in an increase in the polyphenolic yield, while a further increase in the solvent volume (a 40:1 mL/g ratio) did not cause the additional release of polyphenols. One-way ANOVA and Duncan’s post hoc test also showed that a 30:1 mL/g ratio provided the highest TPC, followed by 40:1 and 20:1 mL/g (Figure S1). In HAE, UAE, and MAE, similar values of polyphenol content were determined at 30:1 and 40:1 mL/g ratios (Figure 1A,B), hence statistical analysis of the obtained data showed that there was no statistically significant difference between the mentioned ratios, whereas a 20:1 mL/g ratio gave significantly lower TPCs (Figure S1). The maximal polyphenol content in *F. officinalis* dust extracts was achieved at 30:1 and 40:1 mL/g ratios; therefore, they were chosen for further full factorial design.

Figure 1B,C show that a longer extraction time provided a higher TPC in all used extraction procedures. Nevertheless, in M, HAE, and UAE, it can be observed that the plateau was reached after 60 min, 30 min, and 15 min, respectively, whereas in MAE, the extraction time longer than 120 s caused a decrease in the TPC. One-way ANOVA followed by Duncan’s post hoc test confirmed that time significantly affected the recovery of polyphenols (Figure S1). A significantly higher polyphenol yield was reached after 60 and 90 min of M, 30 and 45 min of HAE, 15 and 30 min of UAE, and 120 s of MAE. On the other hand, the extracts prepared after 30 min of M, 15 min of HAE, 5 min of UAE, and 60 s of MAE possessed significantly lower TPC (Figure S1). In addition, a significant drop in the TPC values after 180 s of MAE in comparison to the parallels obtained after 120 s can be noticed. Since the highest polyphenolic concentrations in fumitory extracts were determined after 60 and 90 min in M, 30 and 45 min in HAE, 15 and 30 min in UAE, and 120 s in MAE, while the TPC was higher after 180 s of MAE than after 60 s, the mentioned levels were selected for future 2^3^ full factorial design.

### 2.2. Experimental Design for Optimization of the Extraction

The experimental design (2^3^ full factorial design) was used to determine the optimal conditions for each tested extraction technique for reaching fumitory extracts with the highest TPC. Following the results of the preliminary screening (Section 2.1), two promising levels of each investigated parameter—ethanol content (50 and 70%), solvent-to-solid ratio (30:1 and 40:1 mL/g), and time (60 and 90 min for M, 15 and 30 min for HAE, 15 and 30 min for UAE, and 120 and 180 s for MAE)—were included in the 2^3^ experimental design. The effects and corresponding regression coefficients of the parameters were determined; the data are shown in Table 1. The observed and predicted TPC values are presented in Table 2.

Full factorial design (2^3^ experimental design) was carried out to investigate the impact of extraction conditions as independent variables on the polyphenol yield of the extracts as the dependent variable. Thus, Table 1 presents all the tested parameters (at the two most promising levels) that can influence the polyphenol content in all four employed extraction procedures.

In M, ethanol content (factor 1) was the only relevant parameter for the TPC of fumitory extracts, while effect and effect estimates had negative values indicating that the lower used level of the parameter (50% ethanol in this case) gave a significant higher polyphenol yield (Table 1). Solvent-to-solid ratio (factor 2) and extraction time (factor 3) did not have a significant effect due to the results from the experimental design (Table 1). Thus, in order to reach the highest TPC, with a reduction in solvent consumption for a shorter extraction time, the optimal conditions for the maceration process of fumitory polyphenols were 50% ethanol as an extraction medium, a 30:1 mL/g ratio, and 60 min. In HAE, all three factors at two levels were not significant for achieving the highest polyphenol content (Table 1). Following the industrial requirements (reduced amount of organic solvent, lower consumption of the extraction medium, shorter extraction time, and higher extraction yield), the optimal conditions for the extraction of fumitory polyphenolic compounds at a high temperature were 50% ethanol, a 30:1 mL/g ratio, and 30 min. In UAE, ethanol content (factor 1) and solvent-to-solid ratio (factor 2) were significant parameters in the extraction of polyphenols from *F. officinalis* dust (Table 1). The effect and effect estimates of both independent variables had negative values, proving that a lower tested level of the parameters (50% ethanol and 30:1 mL/g) provided significantly higher TPC. On the other hand, extraction time (factor 3) was not relevant in the experimental design. The optimal conditions for the UAE of polyphenols from the fumitory were 50% ethanol, a 30:1 mL/g ratio, and 15 min. In MAE, only extraction time (factor 3) was a significant parameter for reaching the highest TPC, whereas effect and effect estimates had negative values, meaning that a lower level of the mentioned factor (120 s in this case) gave a better result, i.e., a higher polyphenol yield. Experiment design has also shown that ethanol content (factor 1) and solvent-to-solid ratio (factor 2) did not have a significant influence on the TPC in MAE. Therefore, the optimal conditions to extract fumitory polyphenols in MAE were 50% ethanol, a 30:1 mL/g ratio, and 120 s of irradiation.

The conclusion on the optimal extraction conditions for all four tested procedures is confirmed by the measured and predicted values of the TPC shown in Table 2.

The measured values under the previously mentioned optimal conditions for M, HAE, UAE, and MAE were 16.56 ± 0.25, 18.33 ± 0.70, 19.04 ± 1.06, and 22.95 ± 1.02 mg GAE/g, respectively (Table 2). The model has predicted the highest polyphenol yield in fumitory extracts under the same conditions for M, HAE, UAE, and MAE (16.61 ± 0.50, 18.41 ± 0.48, 19.12 ± 1.18, and 22.87 ± 0.86 mg GAE/g, respectively) (Table 2). Additionally, it can be concluded that MAE provided the extracts with the highest polyphenol content.

### 2.3. Total Flavonoid, Condensed Tannin, and Protein Contents

The selected four samples (the extracts prepared under the optimal extraction conditions to achieve the highest TPC using all tested extraction procedures) were also characterized in terms of total flavonoid, condensed tannin, alkaloid, and protein contents, and the data are shown in Table 3.

As can be seen in Table 3, the highest flavonoid yield was determined in the fumitory extract obtained in MAE (12.21 ± 0.29 mg CE/g). The extract prepared using HAE possessed a significantly higher TFC (8.50 ± 0.23 mg CE/g) in comparison with the samples from M and UAE (7.09 ± 0.38 and 7.51 ± 0.36 mg CE/g, respectively). The highest condensed tannin content was measured in the extract from MAE (755.0 ± 18.3 µg CE/g), followed by the extract obtained in HAE (715.9 ± 11.8 µg CE/g), M (461.7 ± 18.9 µg CE/g), and UAE (361.6 ± 15.1 µg CE/g) (Table 3). The highest protein yield was determined in the extract obtained in MAE (6.50 ± 0.19 mg AE/g) > UAE (5.77 ± 0.35 mg AE/g) ≥ HAE (5.39 ± 0.22 mg AE/g) ≥ M (5.07 ± 0.30 mg AE/g).

### 2.4. Qualitative and Quantitative LC-MS Analysis and Total Alkaloids of the Extracts

The spectral data and the results of quantitative analyses of fumitory dust extracts are given in Table 4 and Table 5, respectively, while the structures of the identified components and chromatograms are shown in Supplementary Material Figures S2 and S3, respectively.

By analyzing the hydroethanolic extracts of fumitory aerial flowering parts using LC-MS, 21 components were detected, belonging to three different classes of plant secondary metabolites: isoquinoline alkaloids, flavonoids, and phenolic acids. Regarding qualitative and quantitative analysis, four compounds were identified and quantified using authentic commercial standards: chlorogenic acid, quercetin 3-*O*-rutinoside, quercetin-3-*O*-glucoside, and quercetin. Alkaloids were quantified as total fractions using the gravimetric method.

Alkaloids: Seven isoquinoline alkaloids were registered in the investigated extracts. Tentatively (identification level 3 according to Schymanski et al. [32]), five of them (**7**, **11**, **13**, **19**, and **20**) were characterized as alkaloids of the protopine type (protopine, oxo-, methyl and/or acetyl protopine derivatives, and cryptopine), and two of the spirobenzylisoquinoline type (fumariline—**17** and fumarophycine—**18**), based on similarities between the mass and UV spectra and the literature data [30,31]. In the MS of protopine (**13**), the protonated molecule [M + H]^+^ has been registered at *m*/*z* 354, which generated fragment ions at *m*/*z* 336 formed mainly by the neutral loss of H_2_O and *m*/*z* 323 by the loss of the NHR_1_R_2_ moiety from the protonated molecule, while at *m*/*z* 181 and 165 by *α*-cleavage of the skeleton. The characteristic fragment ions that corresponded to the RDA (Retro-Diels–Alder) reaction mainly appear at *m*/*z* 206, fragmenting to ions at 189, 165, and 149 [33].

Flavonoids: The UV spectra with two absorption bands (band I maximum in range 240–260 nm; band II maximum 350–360 nm) indicated the quercetin structure [34] of 11 detected flavonoids (**1**, **3**, **5**, **6**, **8**–**10**, **12**, **15**, **16**, and **21**). Among signals of deprotonated molecules, their MS contained the signals of a deprotonated molecule of quercetin as aglycone at *m*/*z* 301, generated by the neutral loss of a corresponding sugar unit (162 Da from hexose; 146 Da from deoxyhexose; and 132 Da from pentose or adequate diglycoside group), as well as the fragment ions corresponding to RDA reactions of the aglycone part [35]. Quercetin 3-*O*-rutinoside (**8**; [M–H]^−^ at *m*/*z* 609), quercetin 3-*O*-glucoside (**10**; [M–H]^−^ at *m*/*z* 463), and quercetin (**21**) were identified using authentic standards (based on Rt values and UV and MS spectral data). According to the fragmentation patterns of the rest of the detected quercetin glycosides, the structure of **1** corresponded to quercetin trihexoside (deprotonated molecule registered at *m*/*z* 787, fragmenting ions at *m*/*z* 625, 463, and 301); that of **3** to quercetin deoxyhexosyldihexoside ([M–H]^−^ at *m*/*z* 771, giving ions at *m*/*z* 625, 463, and 301); that of **5** to quercetin dihexoside ([M–H]^−^ at *m*/*z* 625); and that of **6** to quercetin pentosylhexoside ([M–H]^−^ at *m*/*z* 595). The MS spectral data of **9**, **12**, **15**, and **16** were equal to the following methylquercetin heterosides: methylquercetin dihexoside (**9** and **16**; [M–H]^−^ at *m*/*z* 639) and methylquercetin pentosylhexoside (**12**; [M–H]^−^ at *m*/*z* 609). Compound **14** was described tentatively as kaempherol deoxyhexosylhexoside ([M–H]^−^ at *m*/*z* 593).

Phenolic acids: Chlorogenic acid (**2**) was identified using the standards. Following UV spectra of hydroxycinnamic acid and MS fragmentation, with [M–H]^−^ at *m*/*z* 295 fragmenting to 179, 135, and 115, the structure of **4** was assigned to caffeoylmalic acid.

Considering the twelve flavonoid metabolites observed overall, quercetin and its five heterosides, as well as three methylquercetn biosides and one kaempferol glycoside, were detected in all four extracts. Quercetin trihexoside (**1**), and methylquercetin pentoside hexoside (**12**) were detected in three tested extracts, except the sample from the microwave reactor. Chlorogenic (**2**) and caffeoylmalic acids (**4**) were identified in all analyzed extracts. Four compounds were identified and quantified using authentic commercial standards: chlorogenic acid (**2**), quercetin 3-*O*-rutinoside (rutin—**8**), quercetin-3-*O*-glucoside (isoquercitrin—**9**), and quercetin (**21**). Rutin (**8**) was additionally used for the quantification of all other detected quercetin biosides and triosides and for methylquercetin glycosides. Compounds **4** and **14** were expressed as caffeic acid and kaempferol 3-*O*-glucoside. The most dominant flavonoids in all four samples were quercetin pentoside hexoside (**6**: from 0.68% in HAE to 0.72% in macerate) and rutin (**8**: 0.70% in HAE to 0.75% in MAE). The total alkaloid fraction (obtained by the gravimetric method) was two- to about three-fold abundant (from 6.885% in macerate to 9.322% in MAE) in comparison to the total phenolic fraction (Table 5). In addition, total alkaloid content was higher in UAE and MAE samples compared to macerate and HAE extracts.

Isoquinoline alkaloids previously identified in ethanol extracts of fumitory herb overall included protopine, cryptopine, fumaricine, dihydrofumariline, fumaritine, O-methylfumarophycine, fumarophycine, fumariline, parfumidine, parfumine, O-methylfumarofine, bicuculline, corlumine, stylopine, sinactine, cheilanthifoline, N-methylcorydaldine, and corydamine [19,20]. Among phenolics, a few phenolic acids, i.e., chlorogenic, *p*-coumaric, ferulic, sinapic acid, and caffeoylmalic acid, were detected earlier in small amounts in ethanol extracts of herb (up to 0.23%). Namely, flavonoids were qualitatively, as well as quantitatively, more abundant phenolics, especially flavon-3-ols, including kaempferol, quercetin, quercetin glycosides (rutin, isoquercitrin, hyperoside, and quercitrin), and myricetin. Rutin was the predominant glycoside (up to 0.65%) in various studies [17,18,21]. Sporadically, flavones or flavanone glycosides were detected [21].

### 2.5. Antioxidant Capacity of Fumitory Extracts

**Figure 2:**
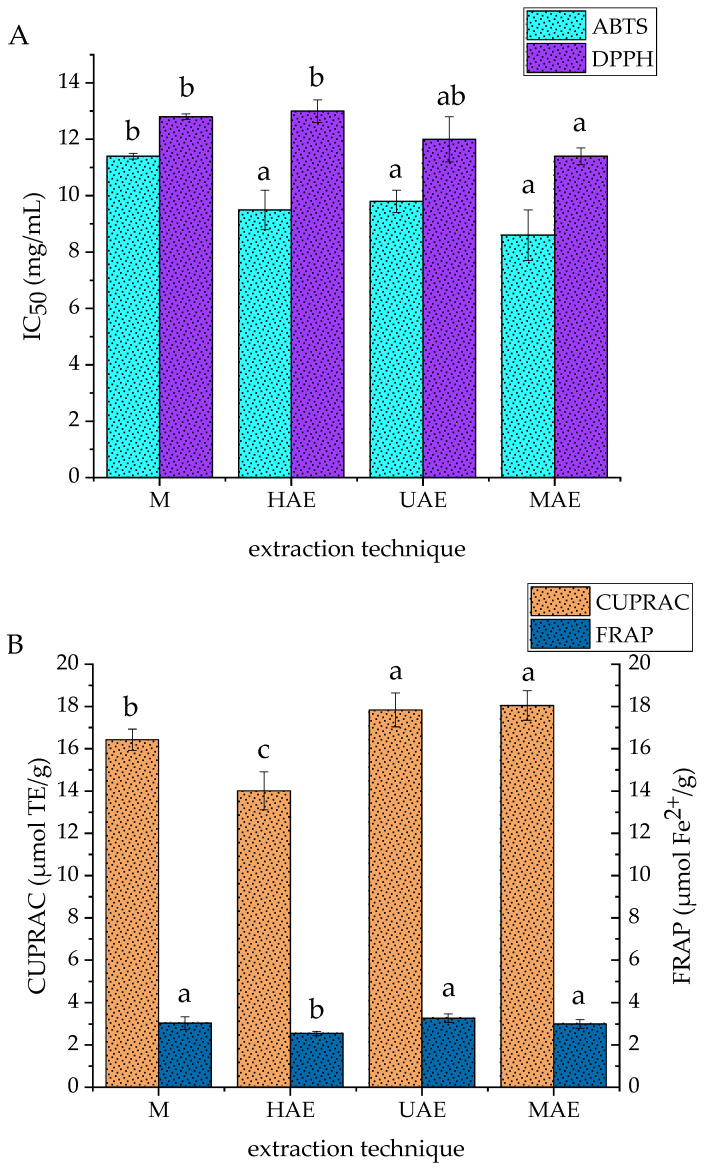
Antioxidant capacity of four selected *Fumaria officinalis* dust extracts obtained in maceration (M) and heat-, ultrasound-, and microwave-assisted extractions (HAE, UAE, and MAE, respectively). (**A**) ABTS and DPPH radical scavenging assays and (**B**) cupric ion-reducing antioxidant capacity (CUPRAC) and ferric-reducing antioxidant power (FRAP) assays. IC_50_, the concentration of the extract necessary to scavenge 50% of free ABTS or DPPH radicals; TE, Trolox equivalent; different letters represent statistically significant differences between the extracts from all extraction techniques (for each antioxidant assay separately) based on one-way analysis of variance and Duncan’s post hoc test (*p* < 0.05, n = 3, mean ± standard deviation).

The antioxidant potential of four fumitory extracts prepared under the optimal extraction conditions using M, HAE, UAE, and MAE, was tested using the ABTS, DPPH, CUPRAC, and FRAP methods, and the results are presented in Figure 2.

As can be seen from Figure 2A, fumitory macerate possessed a significantly lower ABTS radical scavenging capacity (the highest IC_50_ value, 11.4 ± 0.1 mg/mL) in comparison to the other three extracts, whose values varied in a narrow range (8.6–9.5 mg/mL). However, for the DPPH radical scavenging potential (Figure 2A), the trend was different: MAE (11.4 ± 0.3 mg/mL) ≥ UAE (12.0 ± 0.8 mg/mL) ≥ M and HAE (12.8 ± 0.1 and 13.0 ± 0.4 mg/mL, respectively). In the CUPRAC assay, the trend was as follows: UAE and MAE (17.84 ± 0.85 and 18.05 ± 0.71 µmol TE/g, respectively) > M (16.43 ± 0.45 µmol TE/g) > HAE (14.1 ± 0.89 µmol TE/g) (Figure 2B). Regarding the results of the FRAP method, there was no statistically significant difference in the ferric ion reduction between the M, UAE, and MAE extracts (3.00–3.27 µmol Fe^2+^/g), whereas the HAE extract showed a significantly lower potential (2.55 ± 0.10 µmol Fe^2+^/g) (Figure 2B).

### 2.6. The Influence of Fumitory Extracts on HaCaT Cell Viability

**Figure 3:**
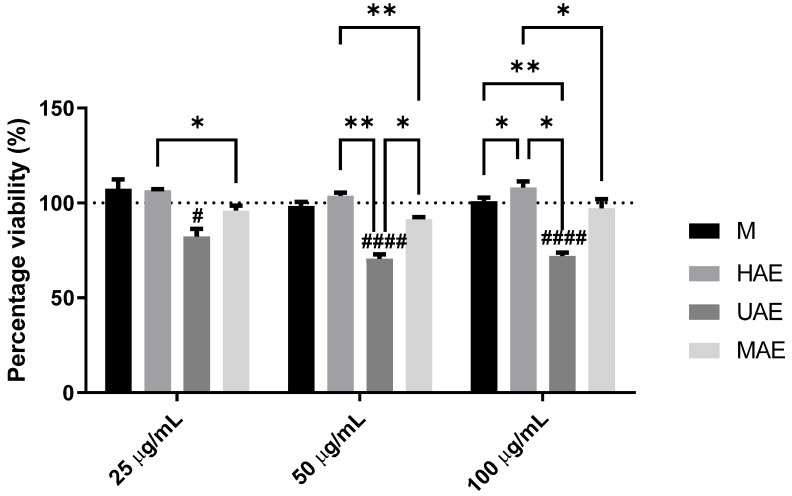
Cytotoxicity of *Fumaria officinalis* extracts in a range of concentrations (25, 50, and 100 µg/mL) determined by the MTT assay in the HaCaT cells. Data are expressed as mean ± standard error of mean relative to the unexposed control (dashed line: # *p* < 0.05 and #### *p* < 0.0001) and multiple comparisons among the extracts (* *p* < 0.05 and ** *p* < 0.01) by one-way analysis of variance (ANOVA) with Tukey’s multiple comparison post hoc test. M—maceration and HAE, UAE, and MAE—heat-, ultrasound-, and microwave-assisted extractions, respectively.

Some alkaloids from *Fumaria* species and their extracts have shown cytotoxic activities [11], thus, selected *F. officinalis* extracts (25, 50, and 100 µg/mL) were examined in terms of keratinocyte viability, and the results are presented in Figure 3.

Figure 3 represents the examination of the cytotoxic potential of fumitory extracts on the HaCaT cells treated for 24 h. Incubation of cells with the extracts for 24 h did not lead to a significant change in cell viability compared to unexposed control. The observed effect was similar for all three concentrations. On the other hand, the UAE extract showed a marked decrease in the percentage of live cells concerning the cells exposed to the medium alone (control). The cytotoxic effect of the UAE sample was concentration-dependent, where a greater reduction in cell viability was observed with increasing concentrations.

### 2.7. The Influence of Fumitory Extracts on the Production of Reactive Oxygen Species in HaCaT Cells

**Figure 4:**
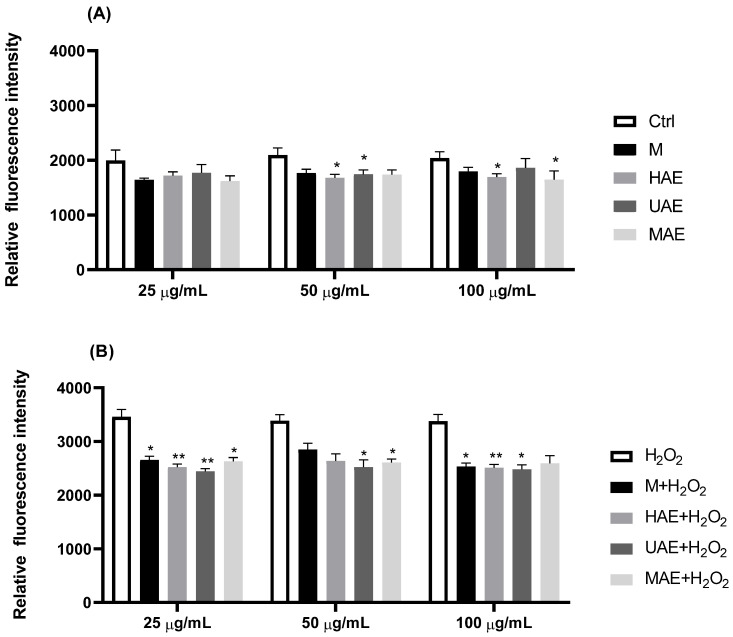
Effect of 24 h pre-incubation with *Fumaria officinalis* extracts in a range of concentrations (25, 50, and 100 µg/mL) on the production of reactive oxygen species (ROS) in the HaCaT cells. (**A**) without H_2_O_2_ and (**B**) after the exposure to H_2_O_2_, determined by H2DCFDA assay, expressed as relative fluorescence intensity. Data are expressed as mean ± standard error of the mean; * *p* < 0.05, ** *p* < 0.01 vs. control (by one-way analysis of variance (ANOVA) with Tukey’s multiple comparison post hoc test). Ctrl, control; M, maceration; HAE, UAE, and MAE, heat-, ultrasound-, and microwave-assisted extractions, respectively.

The impact of selected fumitory extracts (25, 50, and 100 µg/mL) on the production of ROS in keratinocytes was investigated as well; the results are shown as graphs in Figure 4.

Figure 4 represents the effects of *F. officinalis* extracts on the production of ROS in HaCaT cells following the 24-h incubation period. Analysis of the effects of extracts on the levels of ROS before exposure to H_2_O_2_ (Figure 4A) showed that all tested extracts at three selected concentrations underwent a small decrease in endogenous ROS production after 24 h of incubation in HaCaT cells. Incubation with HAE extract at the concentration of 50 µg/mL and 100 µg/mL led to a significant decrease in ROS compared to the untreated control. Also, the UAE sample at 50 µg/mL and the MAE sample at 100 µg/mL significantly reduced ROS production in cells (Figure 4A).

After exposure to 200 µM H_2_O_2_ for 2 h, the production of ROS was elevated in HaCaT cells (H_2_O_2_ bar in Figure 4B). The cells pre-incubated with fumitory extracts showed decreased ROS levels compared to cells exposed to H_2_O_2_ alone in all examined concentrations. Extracts from M and HAE showed a significant decrease at 25 µg/mL and 100 µg/mL, while at a 50 µg/mL concentration, they did not show significant effects due to deviations (Figure 4B). The UAE extract displayed a significant ROS decrease at all examined concentrations. Finally, the extract from MAE showed a significant reduction in H_2_O_2_-induced ROS production in the cells pre-incubated with 25 µg/mL and 50 µg/mL of the MAE sample, while the decrease exhibited at 100 µg/mL was insignificant.

### 2.8. Anti-Inflammatory Effects of Fumitory Extracts

**Figure 5:**
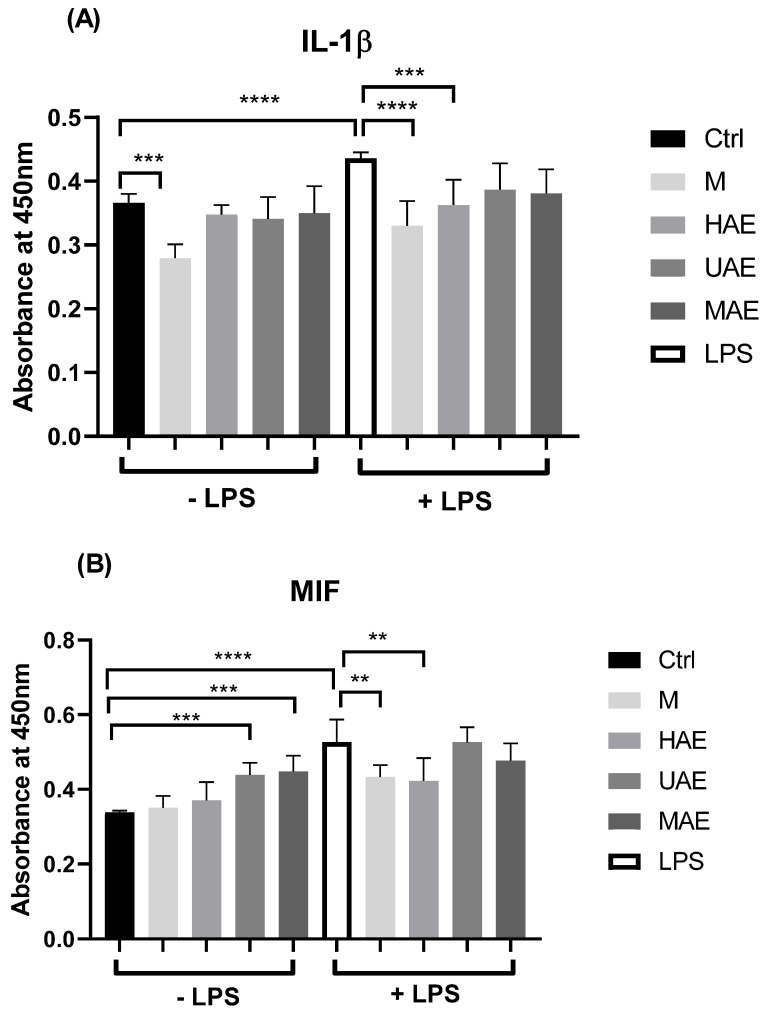
The influence of *Fumaria officinalis* dust extracts from maceration (M) and heat-, ultrasound-, and microwave-assisted extractions (HAE, UAE, and MAE, respectively) on the inflammation reaction caused by bacterial lipopolysaccharide (LPS) on HaCaT cells: (**A**) IL-1β, interleukin 1 beta; (**A**) MIF, macrophage inhibitory factor; Ctrl, control (the cell without extract and LPS); ** *p* < 0.01, *** *p* < 0.001, and **** *p* < 0.0001 (Kruskal–Wallis test with Dunn’s post hoc test).

The *in vitro* anti-inflammatory potential of selected *F. officinalis* dust extracts was investigated using the model of inflammation of the HaCaT cells and the release of IL-6 and MIF induced by bacterial LPS. The data related to the impact of extracts on the inflammation reaction caused by bacterial LPS on the HaCaT cells are presented graphically in Figure 5.

The cytokines IL-1β and MIF are known to play important roles in the regulation of allergic disorders and skin inflammation [36]. LPS is a bacterial endotoxin used to induce local inflammation in skin cells. Thus, the potential anti-inflammatory effects of four selected fumitory dust extracts at a final concentration of 100 µg/mL on keratinocyte cells challenged with LPS were examined. The results presented in Figure 5 show that in cells treated with extracts without LPS, there was a moderate inhibition of IL-1β expression in the HaCaT cells treated with macerate, and a significant increase in MIF by UAE and MAE extracts, indicating their pro-inflammatory effect. Next, LPS treatment alone induced significant elevation of both IL-1β and MIF in exposed cells compared to non-treated controls. In LPS-treated cells, M and HAE extracts significantly reduced IL-1β and MIF expression when pre-incubated for 24 h, compared to LPS alone, confirming the extract’s anti-inflammatory effect against LPS challenge. On the contrary, other types of extracts, such as UAE and MAE samples, did not show a significant change in the level of these pro-inflammatory cytokines after LPS induction.

## 3. Discussion

The impact of various extraction conditions in different employed extraction techniques on the TPC of fumitory dust extracts was diverse. Thus, each extraction factor’s influence has been discussed in detail. The fact that the addition of ethanol in the surrounding water led to the increase in polyphenol yield in all employed extraction procedures can be explained by the changed solvent polarity. Namely, changing the composition of the extraction medium results in its polarity changing, which affects the release of bioactive components into the extraction surroundings, and consequently the biological capacity of the extract [37]. With the aim of avoiding the use of organic solvents and fully adapting to the strict international regulations for their usage [38], in the present study, water as a unique extractant was investigated as well. The adoption of water as an extraction medium is an important step forward in the affordability, profitability, and sustainability of waste management [38]. However, the mentioned extraction solvent did not give a satisfactory polyphenol yield from fumitory dust. Also, according to the literature, higher ethanol amounts in the extraction medium can provide a higher extraction yield, but not always a higher total polyphenol content or biological activity [37]. Besides that, the Khamtache-Abderrahim et al. study [39] has shown that solvent type significantly affected phenolic recovery in fumitory, where 50% ethanol provided a higher TPC in comparison to water, as in the case of the present study. Nevertheless, in the mentioned previous research [39], the highest level of TPC was achieved by employing 50% methanol as an extraction solvent. Since the aim of the present study was the potential dermal application of fumitory dust extracts, methanol was not employed as an extractant, because of its high toxicity. Namely, as a non-GRAS (Generally Recognized As Safe) solvent, methanol is considered a contaminant, biologically aggressive, and consequently inadequate in pharmaceutical, cosmetic, or dermo-cosmetic formulations, making it unfeasible for usage involving contact with humans [38]. On the other hand, ethanol, water, or hydro-ethanol mixtures are commonly accepted as GRAS, and these extraction solvent systems have been successfully used in green extraction procedures of polyphenolic compounds from a wide variety of herbal by-products [38,40]. Ethanol is considered a low-toxicity and environmentally friendly extraction medium that provides a satisfactory polyphenol yield and can be used for large-scale technologies [38,41]. In order to maximize extractability and selectivity, the choice of the extraction medium in MAE should be cautiously performed, due to the expectation for the extractant to combine the solvation capacity of the targeted compounds and heat absorption [42]. The effects of UAE facilitate the efficient release of polyphenols in the presence of ethanol, water, and their mixtures, as GRAS mediums, providing the most efficient mobilization of polyphenols using equivolumetric ethanol–water [38,43]. In MAE, the widely employed mediums include water, methanol, and ethanol, as polar solvents whose dielectric constant provides the absorption of microwave radiation and rapid heating up, consequently reducing extraction periods and preventing a negative impact on thermosensitive components [38,44]. Several studies have shown that efficient MAE used equivolumetric ethanol–water as a medium for extracting polyphenol compounds, including flavonoids, phenolic acids, and anthocyanins from various plant materials [20,45,46].

Solvent-to-solid ratio showed a significant effect on the TPC of fumitory extracts in all tested extraction procedures: 30:1 mL/g ≥ 40:1 mL/g ≥ 20:1 mL/g. According to the literature data, a high solvent-to-solid ratio was found to be favorable in polyphenol extraction, which is consistent with mass transfer principles: high solvent-to-solid ratio = increased concentration gradient = enhanced diffusion rate = greater extraction of species from solids by solvent. Namely, the driving force for mass transfer is the concentration gradient among the extraction surroundings (solvent) and plant matrix (solid) [47,48]. Additionally, the chance of bioactives to contact the extraction medium expands with the increased amount of extractant. However, the increased release of target compounds is not able to be continued once equilibrium is reached [49], which was also the case for fumitory dust extracts (the absence of statistically significant differences in the TPC between 30:1 and 40:1 mL/g). In the case of a higher content of plant material, the rise in viscosity and lower polyphenol diffusion in the extraction solvent caused a lower polyphenol yield in the fumitory extract [29]. Although the TPC increased with the increase in solvent-to-solid ratio, the increase in polyphenol yield may not be directly proportional. Moreover, the use of a high solvent-to-solid ratio can result in diluted extracts, i.e., a lower TPC [47]. Therefore, the examination of the impact of different ratios is necessary with the aim of achieving the efficient usage of solvent for extracting phytochemicals, avoiding the saturation effect, and lowering solvent waste disposal costs [47,49].

The present study shows that the TPC was significantly affected by extraction time; prolonged time gave a higher polyphenol yield in all tested extraction techniques until the plateau was reached (for M, HAE, and UAE) or TPC started to decrease after a certain time (for MAE). From a recovery point of view, it would be more convenient to perform extraction at a lower temperature for a longer exposure time, whereas, considering the industrial application of the extraction procedure, it would be more acceptable, energetically favorable, and profitable to apply higher temperatures for a shorter time [50]. The reason for the absence of a further increase in the TPC of fumitory macerates after 60 min may lie in the fact that the maximum depletion of the herbal material has occurred, considering that a continuous shaking process was included (several hours are required in the process without shaking [50]), while there were no mechanical or heating effects that could provide the additional release of target components. Namely, in maceration, there are two phases in the release of polyphenol compounds: an initial increase in the polyphenol recovery for the first 15 min and slow recovery after 60 min [51]. In HAE and MAE, under high temperatures, plant tissues are softened, and the weak interactions affect the membranes and walls of the plant cells, allowing for a higher rate of recovery for bioactives, i.e., polyphenols can be easily extracted into the medium [52]. Nevertheless, prolonged exposure time at high temperatures decreases the extraction yield of the TPC, because the high temperature facilitates their oxidation and degradation [53]. Thus, the recommended extraction time in HAE and MAE for fumitory dust was 30 min and 120 s, respectively, to preserve the polyphenolic components. In UAE, polyphenol content increased with the increase in exposure time but had already reached a plateau after 15 min of sonication. Saifullah et al. [54] also showed the positive and significant effect of extraction time on the extraction yield of TPC from lemon tea tree leaves in the UAE.

The values of the TPC in fumitory dust extracts agree with the literature data, where Sofiane and Seridi [55] measured from ~14 mg GAE/g to ~30 mg GAE/g in the extracts of *Fumaria* species. The achieved polyphenol yield in the fumitory dust extracts prepared under optimal extraction conditions in four tested extraction procedures follows the trend: MAE > HAE and UAE > M. The higher TPC in HAE and MAE extracts in comparison to macerates can be explained by high temperatures that cause water evaporation from plant cells, the dehydration of cellulose, and excessive pressure within cells, as well as swelling and consequently the rupture of walls and cells. The mentioned destruction of the plant cell structure increases the capillary-porous properties of the herbal material, as well as the potential of solvent absorption, matrix desorption, and diffusion of extractable species to the medium during a shorter extraction period [38,56,57]. On the other hand, the rapid high/low-pressure oscillations during UAE produce vacuum nano-bubbles at countless nucleation points within the propagation paths. Due to successive compression–rarefaction fluctuations, the nano-bubbles accumulate energy and consequently collapse. The simultaneous implosion of innumerable nano-bubbles provides innumerable effects, i.e., shearing forces (as micro-jets) and shock waves [38,57]. Mechanical energy causes significant physical and chemical repercussions on the plant material, by breaking the cell microstructure and decreasing particle size [58]. In addition, the enhanced surface area and penetration of the extraction medium provide a facilitated mass transfer of the components, i.e., higher extraction efficiency in comparison to the maceration procedure [38].

MAE provides a better extraction yield during a shorter exposure time than conventional extractions, due to the heat and mass transfer that occurs from the plant materials to the walls of the extracting vessel [38]. The above-mentioned fact can explain the highest TFC being measured in the MAE fumitory extract, followed by the HAE sample. Significantly lower flavonoid and tannin yields in the UAE sample in comparison to MAE and HAE extracts may indicate that fumitory flavonoid and tannin compounds are more susceptible to ultrasound irradiation than to a high temperature. Elevated temperatures can enhance the efficiency of the extraction process via cell destruction, resulting in the higher recovery of various compounds into the extraction medium [29]. On the other hand, lower levels of flavonoids and tannins in the UAE sample can lie in the ultrasound wave potential to degrade target species, such as antioxidant compounds, by the generation of free hydroxyl radicals, acting as one of the main disadvantages of the utilization of sonication in the extraction process [29,38]. Due to all previously mentioned mechanisms in the MAE process, the highest protein concentration was expected in the fumitory MAE sample. Namely, the literature data also reported higher protein recovery in the extraction solvent, because of the degradation impact of microwave irradiation on herbal tissue and cell structures [59,60].

The antiradical capacity of fumitory dust extracts prepared using four different extraction procedures was measured via their ability to entrap an unpaired electron of the DPPH radicals or to capture free ABTS radicals [3,61]. In both antiradical assays used, the extraction technique significantly affected the antioxidant potential of the fumitory extracts. A slightly decreased DPPH antioxidant capacity in the UAE sample compared to the MAE parallel can be explained by the sensitivity of natural antioxidants to air exposure and the generation of free radicals by ultrasound waves [59,62]. In MAE, high internal pressure and an elevated temperature enhance the solubility of antioxidants in the extraction solvent, increasing the antioxidant activity of the extracts [59]. According to the literature, the percentage of radical scavenging depends on the chemical nature of the antioxidants and their concentration [3,63]. The anti-DPPH radical potential can be attributed to the presence and position of hydroxyl functional groups in the molecular structure of the antioxidant components, as well as the availability of hydrogen [3]. Khamtache-Abderrahim et al.’s study [3] showed that the alkaloid extract of *F. officinalis* with the lowest alkaloid yield possessed the highest anti-ABTS activity (the lowest IC_50_ value), although the activity was not alkaloid concentration-dependent. In addition, the same study reported that the DPPH radical scavenging potential of the extracts was not alkaloid dose-dependent, which was also shown in fumitory dust extracts. Namely, the position of functional groups and their degree of methylation can have a significant role in the antiradical capacity of alkaloid compounds [3,64]. The ABTS radical neutralization potential of the fumitory extracts can also be explained by a possible synergism among different alkaloid compounds [3]. Nevertheless, the fumitory extract’s potential to neutralize free ABTS and DPPH radicals can be related to the yield of extracted flavonoids and condensed tannins. Specifically, the MAE sample possessed the highest levels of flavonoids and condensed tannins, as well as the lowest IC_50_ values (the highest antioxidant activity) in both antiradical tests. Namely, flavonoids and tannins show strong antioxidant activity. Edziri et al.’s study [11] reported that *F. officinalis* extracts with high antioxidant capacity also possessed high polyphenol and flavonoid yields. According to the literature data, the concentration of earth smoke water extract required to neutralize 50% of the initial ABTS radicals was 96.0 μg/mL, whereas the crude alkaloid extract showed an IC_50_ value of 70.71 ± 0.04 μg/mL [3,13]. The antioxidant activity of the aerial part extracts of *F. officinalis* originating from Tunisia also showed lower IC_50_ values (7.39–29.76 µg/mL) [11] in comparison to the results obtained in the present study. The obtained values for the DPPH radical scavenging potential of fumitory dust extracts agree with the literature, where the IC_50_ of *Fumaria indica* extract was 11 mg/mL [65]. However, in the DPPH assay, Ivanov et al. [21] showed lower IC_50_ values for different *Fumaria* species, ranging from 2.39 to 5.15 mg/mL. Moreover, various *F. officinalis* extracts possessed IC_50_ values in a range from 2.50 to 18.43 µg/mL depending on the employed extraction medium [11]. The extracts prepared using various *Fumaria* species displayed a low and dose-dependent ferric ion-reducing capacity, ranging from 0.205 to 0.390 µg/mL [66]. The ethanol–water and methanol extracts of various parts of *Papaver rhoeas* (the plant from the Papaveraceae family related to the family Fumariaceae) have IC_50_ values in the DPPH test in a range from 0.50 to 4.97 mg/mL, which are lower values (higher antiradical potential) in comparison to the extracts obtained in the present study [67,68]. The study performed by Selen Isbilir and Sagiroglu [69] showed that *P. rhoeas* extracts had IC_50_ values, ranging from 1.39 to 5.49 mg/mL depending on the extraction solvents used. Pharmacological investigations of fumitory have also included the antioxidant potential of alkaloids and phenolic acids derived from the mentioned plant. Alkaloids exhibited superior antioxidant properties in terms of inhibiting tertiary butyl hydroperoxide-induced lipid peroxidation compared to phenolic acids. As anticipated, caffeic and protocatechuic acids demonstrated more potent anti-DPPH activity than alkaloidal compounds [70]. Since the reducing capacity of extracts can be a significant indicator of their antioxidant activity, the mentioned property was determined by the reduction of Cu(II) to Cu(I) and the complex (Fe^3+^) to ferrous ions (Fe^2+^), in the presence of antioxidants. The FRAP method showed that the HAE sample possessed the lowest ion-reducing power, and that there was no statistically significant difference in ion-reducing potential among the other extracts, although their alkaloid yields differed. Khamtache-Abderrahim et al. [3] also reported that the ferric ion-reducing potential of fumitory extracts was not alkaloid concentration-dependent, as in the present research, where the samples with the highest levels of alkaloids did not possess the highest reducing power in the FRAP assay. The observed phenomenon can be explained by the modulation of the intensity of the reducing potential due to the diversity of the antioxidant structures and contents, as well as by the number and position of hydroxyl functional groups [3]. Furthermore, Ivanov et al.’s study [21] showed that *Fumaria* species extracts with lower contents of quercetin derivatives, such as rutin, possessed lower ferric and cupric ion-reducing potential. Hence, fumitory dust extracts from HAE contained the lowest levels of rutin and other quercetin derivatives, including quercetin dihexoside, quercetin pentoside hexoside, and methylquercetin dihexoside, regarding the results of LC-MS analysis, and consequently showed the lowest ferric and cupric ion-reducing capacity. The extracts prepared using various *Fumaria* species displayed a low and dose-dependent ferric ion-reducing capacity, ranging from 0.205 to 0.390 µg/mL [66]. Also, the antioxidant potential of *F. vaillantii* extracts determined in the FRAP assay amounted to 248.96–462.3 μmol Fe^2+^/mg of extract, depending on the phenological stages of the plant. In the research on the *P. rhoeas* extract, the FRAP test result was 1.85 mmol Fe^2+^/g, which is higher than those of fumitory dust extracts, as in the case of antiradical activity. *P. rhoeas* extracts prepared using various extraction mediums showed a high reducing activity, which is a significant indicator of their potential antioxidant capacity [69].

In addition to alkaloids, flavonoids, steroid compounds, and organic acids have also been identified as bioactive compounds in *Fumaria* species, showing analgesic, antiproliferative, antioxidant, and anti-inflammatory potential [2,3,11,17,21,71,72]. These effects are particularly due to the isoquinoline alkaloid compounds in the plant; protopine is the most common [72]. Thus, the fumitory dust extracts’ cytotoxicity and antioxidant and anti-inflammatory potential were examined on the keratinocyte lines. Only the UAE extract showed concentration-dependent cytotoxicity on HaCaT cells. The reason for this may lie in its higher alkaloid content than M and HAE samples, considering that alkaloids can be carriers of toxicity [11]. Although MAE extract possesses a higher alkaloid yield as well, its effect on cell viability was insignificant, probably due to its significantly higher contents of flavonoids and tannins with cell protective properties [73,74,75]. For example, the MAE extract possesses the highest level of rutin, a flavonol with proven cytoprotective potential [75]. Abidi et al.’s study [76] has shown that extracts rich in flavonoids and tannins possess antioxidant and skin-protecting effects. Edziri et al. [11] have investigated the influence of fumitory extract on the viability of human hepatoma cells. However, some of the tested extracts showed cytotoxic effects even at 84 µg/mL. Adham et al.’s [2] study demonstrated the cytotoxicity of fumitory extracts rich in alkaloids and flavonoids on the multiple myeloma cell lines via apoptosis, autophagy, and ferroptosis. Additionally, the viability rate of treating *Leishmania tropica* cells with crude *F. officinalis* extracts increased significantly in all tested concentrations (0.007–0.25 mg/mL). However, there were no effects of the crude *F. officinalis* extracts on the viability rates of L20B cell lines at any of the concentrations used, except for 0.125 mg/mL, which showed a decrease in viability rate [77]. *F. officinalis* leaf extracts showed antiproliferative activity, significantly inhibiting breast cancer cell proliferation [78], while the cytotoxic effect of *F. officinalis* extracts was shown on various human carcinoma cells as well [79]. According to Misiurek et al. [80], *F. officinalis* extract showed the highest cytotoxic activity against human Caucasian malignant melanoma cell lines, and low cytotoxicity against other human malignant melanoma cell lines. Sanguinarine, a representative member of quaternary benzo[c] phenanthridine alkaloids discovered in *Fumaria* species, induced apoptosis in HaCaT human aneuploid immortal keratinocyte cells [81]. The significant difference in comparison to the results obtained in the present study can be explained by the differences in the cell lines and extraction mediums used. Additionally, the Hijazi et al. [82] study has shown that the IC_50_ values of alkaloids from *P. rhoeas* toward the human keratinocyte cell line were 110 µM. The extract of *P. rhoeas* did not show a toxic effect on keratinocytes at concentrations of 100, 250, and 500 µL/mL, whilst the effect on skin cell proliferation was observed, i.e., an increase in proliferation was noticed at 250 µL/mL. *Lamprocapnos spectabilis* herb extract (from *Lamprocapnos* as the sister group to the subfamily Fumarioideae) significantly inhibited the viability of melanoma cells, with an IC_50_ value of 4.13 μg/mL [80]. Thio et al.’s study [83] showed the dose-dependent inhibitory effect of fumaric acid derivatives on keratinocyte proliferation, which is linked to the transient intracellular free calcium concentration elevations, while fumaric acid was the least potent growth inhibitor. Aqueous and methanolic extracts of fumitory exhibited moderate antioxidant activity *in vitro* [84,85]. Thus, the antioxidant potential of fumitory extracts toward ROS in the HaCaT cell line was tested in the absence and presence of H_2_O_2_. All fumitory dust extracts at three concentrations affected a small decrease in endogenous ROS production in HaCaT cells in the absence of H_2_O_2_, while the HAE sample at two used concentrations and UAE and MAE samples at one used concentration showed the highest effect. Only the UAE sample significantly affected the ROS levels in the presence of H_2_O_2_ for all tested doses. This was probably due to the smaller cell number, since these concentrations reduced the cell viability, as shown in the MTT assay. However, the trend cannot be observed in both non-treated and H_2_O_2_-treated HaCaT cells. Flavonoid molecules act as exogenous antioxidant components that are directly oxidized by free radicals to form less reactive species. The potential mechanisms include the inhibition of nitric oxide synthase and xanthine oxidase activity, the modulation of channel pathways, and interaction with other enzymes [74]. Isoquercitrin (identified and quantified in fumitory dust extracts as well) exhibited concentration-dependent antioxidant capacity and a high level of activity in the multi-pathways-based ROS scavenging, as well as effective cytoprotection [86]. Chalcones, as aromatic ketones, are natural precursors of flavonoid and iso-flavonoid compounds that can activate the nuclear factor erythroid 2-related factor (2NRF2) pathway and suppress ROS and oxidative stress, providing significant protection of H_2_O_2_-induced oxidative damage in the cells [74]. Studies have indicated that *Fumaria parviflora* can prevent ROS formation and cell damage, i.e., decrease levels of inflammatory and oxidative stress [87,88,89,90]. On the other hand, *F. officinalis* extracts induced ROS production and DNA damage in cancer cells [2]. Wasu et al.’s [91] study reported that *F. officinalis* extract prevented ethanol-induced changes in the immunological and oxidative stress parameters (the reduction in oxidative stress), and that the effects were comparable to those of vitamins C and E. Todorova et al. [92] reported that *P. rhoeas* extract has high antioxidant potential in the anti-ROS biological assay. *P. rhoeas* extracts can also remove H_2_O_2_ from the reaction medium, showing scavenging activity ranging from 10.75 to 39.8 at concentrations of 250 and 400 μg/mL depending on the extraction medium used [69].

Kaempferol and rutin (presented also in fumitory extracts) are flavonoids with anti-inflammatory effects [74]. Alkaloids are an important class of compounds with an anti-inflammatory capacity via the inhibition of expression of cytokines, lipid mediators, histamine, and enzymes in the inflammatory response [93]. *In vivo* studies of protopine have revealed a broad spectrum of biological activities, including antiarrhythmic, antithrombotic, anti-inflammatory, and hepatoprotective effects [94]. Flavonoid glycosides also show anti-inflammatory activity by inhibiting LPS-induced NO production in a macrophage cell line [95]. Plant extracts with high polyphenol and flavonoid contents that possess anti-inflammatory properties can inhibit IL 1 ά and enzymes involved in inflammation pathways [74]. Therefore, the pro-inflammatory potential of MAE and UAE samples shown on the HaCaT cell line without LPS can be explained by a higher protein content in the mentioned extracts. Namely, plant proteins can have peptides bearing potential pro-inflammatory epitopes, or capable of inducing pro-inflammatory cytokines [96]. Hence, UAE and MAE extracts did not exert anti-inflammatory effects on the HaCaT cells after LPS treatment, probably due to their protein pro-inflammatory potential. The anti-inflammatory potential of M and HAE fumitory extracts on keratinocytes can be attributed to a higher content of tannins in comparison to the UAE sample. Namely, tannins can inhibit IL-1β production by blocking nuclear factor kappa B activation [97]. Additionally, according to the literature data, chlorogenic acid (also present in fumitory extracts, with the lowest amount measured in the MAE sample) can inhibit NO production and the expression of COX-2 and pro-inflammatory cytokines, including IL-1β [98]. Kumar Singh et al.’s study [99] reported that *Fumaria indica* extract also suppressed the elevated level of cytokines, including IL-1β, TNF-α, and IL-10, in animals.

The chemical profile of tested fumitory dust extracts relates to the observed biological activities, particularly for dermatological applications. Namely, along with its high antioxidant potential, quercetin also possesses numerous health-promoting activities on the skin, including anti-inflammatory, anti-psoriasis, wound-healing, skin-whitening, anti-itching, anti-aging, and photoprotective effects [100]. Rutin possesses strong antioxidant efficacy, and an anti-inflammatory, wound healing, and cytoprotective capacity [75,100,101]. Isoquercitrin shows beneficial effects against oxidative stress in skin tissue by decreasing ROS levels and is identified as an anti-inflammatory agent by reducing the level of COX-2 and inflammatory cytokines (IL-6, IL-1β, and TNF-α) [102]. Chlorogenic acid significantly downregulated the expression of inflammatory cytokines by reducing the activity of the NF-κB signaling pathway, showing anti-inflammatory potential in a model of skin inflammation [103], as well as decreasing the ROS level in the skin fibroblast cell line [104]. Additionally, kaempferol can ameliorate oxidative skin pathological symptoms, such as cytotoxicity and inflammation due to the scavenging of intracellular ROS [105]. Protopine also shows anti-inflammatory activity via the inhibition of kappa B alpha phosphorylation in the cytosol, and COX-2 activity [106].

Due to the proven biological effects related to skin health benefits (antioxidant and anti-inflammatory potential and the absence of cytotoxicity on keratinocytes), fumitory dust extracts can be implemented as a promising and potential source of bioactives in dermo-cosmetic and pharmaceutical preparations for dermatological circumstances. However, additional analyses of extracts are necessary, such as the determination of potential pesticide residues, contents of heavy metals, and microbiological safety, followed by future experiments related to the development of various formulations, investigation of their stability under different environmental conditions, and assessment of the shelf life of the products. Testing the effectiveness of preservatives in products using the *challenge* test, dermatological testing, and the examination of products’ efficiency are necessary for product claims.

## 4. Materials and Methods

### 4.1. Herbal Material and Chemicals

The *F. officinalis* plant matrix was herbal dust produced as a result of intensive grinding in an industrial mill (UMČ 30, Biljotehnika, Pančevo, Serbia), and unequal parts of the initial herbal material (including stems, leaves, and flowers) in the production sector of the Institute for Medicinal Plants Research “Dr Josif Pančić”, Pančevo, Serbia (size ≤ 0.3 mm). After sieving through pharmaceutical sieves, the mentioned plant matrix takes the form of waste or dust, and according to regulations on the quality of tea, herbal tea, and tea products of the Republic of Serbia, cannot be an integral part of tea products for the market, trade, or sale [28].

Water was purified through a Simplicity UV^®^ water purification system (Merck Millipore, Merck KGaA, Darmstadt, Germany). The water used for the LC-MS analysis was purified by a TKA water purification system (Niederelbert, Germany). Folin–Ciocalteu reagent, Coomassie^®^ Brilliant blue G 250, phosphoric acid, and gallic acid were from Merck (Darmstadt, Germany); ethanol and sodium carbonate from Fisher Science, Loughborough, UK; sodium nitrite from Alkaloid, Skopje, Macedonia; vanillin, hydrochloric acid, and phosphoric acid from Merck, Rahway, NJ, USA; ammonium acetate and nitric acid (65%) from Zorka Pharma, Šabac, Serbia; 1M sodium hydroxide from Alfapanon, Bački Petrovac, Serbia; 2,2′-azino-bis(3-ethylbenzothiazoline-6-sulphonic acid) or ABTS, 6-hydroxy-2,5,7,8-tetramethylchroman-2-carboxylic acid or Trolox, 2,2-diphenyl−1-picrylhydrazyl or DPPH, phosphate-buffered saline (PBS), sodium dodecyl sulfate (SDS), bovine serum albumin (BSA), tetramethylbenzidine (TMB), aluminum chloride, and acetic acid from Sigma-Aldrich, Darmstadt, Germany; methanol, acetone, bacterial lipopolysaccharide (LPS; *Escherichia coli* 055:B5), catechin, and LC-MS-grade acetonitrile and formic acid from Sigma-Aldrich, Burlington, MA, USA; and potassium persulfate from Centrohem, Stara Pazova, Serbia. DMEM/F12 cell culture medium (1:1 mixture of Dulbecco’s Modified Eagle’s Medium and Ham’s F-12 nutrient mixture) was from Pan-Biotech (Aidenbach, Germany). The study was conducted on the spontaneously immortalized human keratinocyte cell line HaCaT from adult skin (a generous gift from Prof. Milica Pešić, Institute for Biological Research “Siniša Stanković”, Department of Neurobiology, National Institute of the Republic of Serbia, University of Belgrade, Belgrade, Serbia), which has been proposed as a model for the study of keratinocyte functions [107,108]. It displays normal morphogenesis and all major surface markers and functions characteristic of isolated keratinocytes [109]. HaCaT cells were cultured in Dulbecco’s Modified Eagle’s Medium (DMEM)–F12 supplemented with 10% FBS and 1% antibiotic/antimycotic solution (herein—complete culture medium). Cell-permeable oxidation-sensitive probe H2DCFDA (2′,7′-dichlorofluorescin diacetate—Calbiochem) was from Merck Millipore (Darmstadt, Germany), while MTT reagent (thiazolyl blue tetrazolium bromide, 1 mg/mL), and dimethyl sulfoxide (DMSO) were from Sigma-Aldrich (St. Louis, MO, USA).

### 4.2. Cell Culture

HaCaT human keratinocytes were kept in 25 cm^2^ tissue culture flasks in a humidified incubator at 37 °C, with 5% CO_2_. They were grown in a complete medium containing Dulbecco’s Modified Eagle’s Medium/Nutrient Mixture F-12 Ham (DMEM F 12, Biowest, Nuaillé, France), 10% fetal calf serum (FCS, Gibco, Waltham, MA, USA), and 1% antibiotic–antimycotic solution (Capricorn Scientific GmbH, Ebsdorfergrund, Germany). After reaching 70% confluence, the cells were trypsinized (0.25% trypsin–EDTA solution, Institute for Virology, Vaccines, and Serum “Torlak”, Belgrade, Serbia), seeded in 96-well plates (1.5 × 10^4^ cells/well) and let to attach to wells for 24 h at 37 °C, with 5% CO_2_, before the treatment.

### 4.3. Extraction Techniques

#### 4.3.1. Maceration

The maceration procedure was performed at room temperature (25 ± 2 °C) in the incubator shaker KS 4000i control, produced by IKA (Staufen, Germany) using three different extraction times (30, 60, and 90 min), three different extraction solvent types (water, 50% ethanol, and 70% ethanol), and three different levels of solvent-to-solid ratio (20:1, 30:1, and 40:1 mL/g). The Erlenmeyer flasks were covered with aluminum foil to avoid evaporation of the medium and light exposure. After extraction, the samples were filtered through filter paper (fine pore, 0.45 µm) and stored at 4 °C until further experiments.

#### 4.3.2. Heat-Assisted Extraction (HAE)

HAE was performed at 80 °C in the incubator shaker using the same factor levels of solvent type and solvent-to-solid ratio as in the maceration process, while extraction times were different (15, 30, and 45 min). In the preliminary screening, various temperatures were employed (40–80 °C), and the highest value of the polyphenol yield was obtained at 80 °C. Therefore, this temperature was set for future experiments in HAE.

#### 4.3.3. Ultrasound-Assisted Extraction (UAE)

UAE was performed at 25–27 °C in the ultrasound bath (digital ultrasound bath, DU-32, ARGO LAB, Carpi, Italy) with a frequency of 35 kHz. A flask with the sample was continuously cooled by adding ice to the bath during extraction, thus the temperature of the sample was measured and controlled. UAE was performed by employing the same parameter levels as in the previous cases and three different extraction times (5, 15, and 30 min).

#### 4.3.4. Microwave-Assisted Extraction (MAE)

MAE was carried out at 100 °C in a microwave reactor Monowave 300 produced by Anton Paar (Graz, Austria) using a magnetic stirring bar at the speed of 600 rpm, the same extraction mediums and solvent-to-solid ratios as in the previous extraction procedures, and three different extraction times (60, 120, and 180 s). In the preliminary study, different temperatures in the microwave reactor were investigated (60−160 °C) and the highest polyphenol concentration was achieved at 100 °C.

All prepared samples were stored at 4 °C until further experiments.

#### 4.3.5. Lyophilization of the Extracts

The selected liquid *F. officinalis* extracts (obtained under the optimal extraction conditions in all employed extraction techniques, i.e., the samples with the highest polyphenol yield) were lyophilized to prepare samples for LC-MS analysis and the determination of anti-inflammatory potential and effects in the cell culture. Ethanol was evaporated using a vacuum evaporator, Heizbad Hei-VAP (Heidolph, Heidelberg, Germany) at 50 °C and 50 mbar. The samples were frozen at −80 °C for 1 h and freeze-dried in Alpha 2–4 LSCplus freeze dryer (Christ, Osterode am Harz, Germany) at a pressure of 0.011 mbar for 24 h.

### 4.4. Quantification of Phytochemicals in the Extracts

#### 4.4.1. Quantification of the Total Polyphenols

The total polyphenol content (TPC) was determined spectrophotometrically in all prepared extracts to investigate the optimal extraction conditions for reaching the highest polyphenol content. Thus, a modified Folin–Ciocalteu assay was employed [110]. Since gallic acid was used for the calibration curve, the TPC was expressed as milligrams of gallic acid equivalent per g of plant material (mg GAE/g).

#### 4.4.2. Quantification of the Total Flavonoids

The total flavonoid content (TFC) in *F. officinalis* extracts obtained under the optimal extraction conditions in all employed extraction techniques (the samples with the highest polyphenol content) was determined spectrophotometrically using a colorimetric method [111]. Since catechin monohydrate was used for the calibration curve, the TFC was expressed as milligrams of catechin equivalent per g of plant material (mg CE/g).

#### 4.4.3. Quantification of the Condensed Tannins

The condensed tannin content of *F. officinalis* extracts (the same samples as in the analysis of TFC) was determined using the vanillin–HCl method [112]. The extract (50 µL), vanillin in methanol (4%, 1500 μL), and concentrated HCl (750  μL) were mixed. The mixture was incubated for 20 min at room temperature and the absorbance was measured at 550 nm. Since catechin monohydrate was used for the calibration curve, the condensed tannin content was expressed as milligrams of catechin equivalent per g of plant material (mg CE/g).

#### 4.4.4. Determination of the Total Alkaloid Fraction

The total alkaloid fractions were determined by the gravimetric method. The extractions were performed, with ether and the addition of a base and an aqueous solution of HCl, subsequently, to convert alkaloids into the free form, and extracted with ether. After removing the ether, the dry residue was measured [113].

#### 4.4.5. Quantification of the Total Proteins

The total protein content of selected *F. officinalis* extracts (the same extracts as in the case of TFC) was determined in the method described by Bradford [114]. Albumin was used as a standard for the calibration curve and the results were expressed as mg of albumin equivalent per g of plant material (mg AE/g).

All absorbance measurements were carried out in triplicate using the UV Spectrophotometer UV-1800 (Shimadzu, Kyoto, Japan).

### 4.5. LC-MS Analysis

Four extracts of *F. officinalis* herb were qualitatively and quantitatively investigated using the optimized LC-MS method. The analysis was performed on an Agilent LC/MS System 1260/6130 (Agilent Technologies, Waldbronn, Germany), operated with ChemStation software (version number Rev. B.04.03-SP1), using Zorbax SB-Aq column (150 × 3.0 mm, 3.5 µm particle size). The operating solution concentration of the tested extracts was 5 mg/mL after being filtered through a 0.45 µm cellulose membrane filter. The mobile phase, operating conditions, and Mass Selective Detector (MSD) ion source parameters were described in Elferjane et al.’s study [115]. Diode Array Detector (DAD) spectra were recorded at 210, 254, 320, and 350 nm, while mass spectra (MS) were acquired in negative and positive ion source modes using the full-scan quadrupole mode (*m*/*z* 80–1500).

By comparing their retention times (Rt), UV spectra, and MS with commercially available standards, four compounds were identified. The structures of other detected compounds were assigned with the highest possible confidence based on UV and/or MS, comparing them with the published data. Quantification was conducted using the external standard method, with calibration curves of five standards. Concentrations of the detected compounds were determined from the peak areas obtained from DAD (at 320 and 350 nm). The regression equations, correlation coefficients (r^2^), linear ranges, and limits of detection (LOD) and quantification (LOQ) are summarized in Table S1. LODs and LOQs were determined based on signal-to-noise ratios of 3 and 10, respectively, following the guidelines of the International Conference on Harmonization [116].

LOD = 3.3 × σ/a(1)

LOQ = 10 × σ/a(2)

### 4.6. Investigation of the Antioxidant Capacity of the Extracts

The antioxidant capacity of selected *F. officinalis* extracts was investigated by employing four antioxidant assays: ABTS, DPPH, CUPRAC, and FRAP tests.

#### 4.6.1. ABTS Test

The ABTS method was employed to investigate the radical scavenging potential of fumitory extracts [117]. The percentage of radical neutralization was calculated using the following equation:

% neutralization = (A_ABTS_ − A_x_) × 100/A_ABTS_ (3)

where A_ABTS_ was the absorbance of ethanol ABTS solution and the extraction medium, and A_x_ was the absorbance of ethanol ABTS and fumitory extract. The antioxidant capacity was expressed as the concentration of fumitory extract required to neutralize 50% of the ABTS radicals, IC_50_ (mg/mL). The IC_50_ value was calculated from the plotted graph of the ABTS radical scavenging activity and different concentrations of the extract.

#### 4.6.2. DPPH Test

The DPPH method was used to examine the radical scavenging capacity of fumitory extracts too [24]. The percentage of radical inhibition was calculated using the following equation:

% inhibition = (A_DPPH_ − A_x_) × 100/A_DPPH_ (4)

where A_0_ was the absorbance of ethanol DPPH solution and the extraction medium, and A_x_ was the absorbance of ethanol DPPH solution and fumitory extract. The antioxidant capacity was expressed as the concentration of fumitory extract required to neutralize 50% of the DPPH radicals, IC_50_ (mg/mL). The IC_50_ value was calculated from the plotted graph of the DPPH radical scavenging potential and different concentrations of the extract.

#### 4.6.3. CUPRAC Test

The cupric ion-reducing antioxidant capacity of fumitory extracts was tested according to the procedure described by Dziurka et al. [118]. Trolox was used as a standard for the calibration curve. The results were expressed as µmol Trolox equivalent (TE) per g of plant material (µmol TE/g).

#### 4.6.4. FRAP Test

The ferric-reducing antioxidant potential of fumitory extracts was also investigated using the method described by Rajurkar et al. [119]. FeSO_4_ was used as a standard for the calibration curve. The results were expressed as µmol Fe^2+^ equivalents per g of plant material (µmol Fe^2+^/g).

### 4.7. Assays on Cell Culture

#### 4.7.1. Treatment Preparation

Stock solutions of *F. officinalis* extracts (M, HAE, UAE, and MAE) were prepared in DMSO at a concentration of 100 mg/mL, and kept at 4 °C. For the experiment, final concentrations of each extract were prepared from the stock solution by dissolving in fresh complete cell medium to reach final concentrations of 25, 50, and 100 µg/mL. These concentrations were further used for cell treatments.

#### 4.7.2. Cytotoxicity Evaluation

The influence of four selected fumitory extracts on the viability of keratinocytes was investigated using the previously described method [120]. The HaCaT cells in complete RPMI medium were seeded in 96-well plates at a density of 1.5 × 10^4^ cells/well, in a final volume of 100 µL per well. The medium was exchanged after 24 h, and treatments were added in a total volume of 100 µL/well. Following incubation with the treatments or solvent (DMSO vehicle control containing matched DMSO concentration) at 37 °C for 24 h, an MTT assay was performed. All other experimental conditions were previously optimized according to the recommendations for optimization of cell viability assays [121] (e.g., cell seeding density) to be cell line specific, and they had minimal effects on the dose–response curves. MTT reagent was added (10 µL per well), and the cells were left for 2 h in the dark at 37 °C for the reaction to occur. Further, purple formazan crystals were dissolved with SDS. Finally, the absorbance was measured at 570 nm on a microplate reader (Epoch, BioTek, Winooski, VT, USA) after the complete solubilization of the crystals. Data were expressed as percentage viability concerning control (100%). Mean values were represented on bars, from three independent experiments performed in triplicate (n = 9).

#### 4.7.3. H2DCFDA Assay (2′,7′-Dichlorofluorescin Diacetate)

The antioxidant impact of four selected fumitory extracts in the HaCaT cell line was examined as well [122]. HaCaT cells were left overnight to attach to the wells and kept in a humified incubator at 5% CO_2_ and 37 °C. The next day, the medium wash was exchanged and fumitory extracts (M, HAE, UAE, and MAE) at final concentrations (25, 50, and 100 µg/mL) in complete medium were added to the cells (100 μL per well). After 24 h, treatments were removed, and cells were rinsed with PBS. Next, the assay was performed in line with the manufacturer’s instructions. Using PBS as the diluent, 5 μM of the cell-permeable oxidation-sensitive probe H2DCFDA was added to the cells and left for 45 min in the dark. Next, the cells were washed with PBS and exposed to PBS alone (control) or the 200 μM H_2_O_2_ used as a positive control. After an incubation time of 2 h, and the conversion of non-fluorescent H2DCFDA to the highly fluorescent 2′,7′-dichlorofluorescein (DCF), the level of generation of intracellular ROS (reactive oxygen species) in cells was determined by measuring the fluorescence on a fluorescent plate reader (Wallac 1420 multilabel counter Victor 3V, PerkinElmer Life and Analytical Sciences, Boston, MA, USA) at excitation and emission wavelengths of 485 and 535 nm, respectively. The data were expressed as relative fluorescence intensity, and the mean value was represented in figures from three independent experiments performed in triplicate (n = 9).

#### 4.7.4. Cell-Based ELISA

The determination of interleukin 1 beta (IL-1β) and macrophage inhibitory factor (MIF) expression was examined by employing the CELISA (cell-based ELISA) method according to the previously described test [123]. Namely, HaCaT cells were seeded in 96-well plates at a density of 2 × 10^5^ cells per well and grown for 24 h at 37 °C and 5% CO_2_. The following day, the medium was replaced with treatments containing fumitory extracts at a final 100 µg/mL concentration in a complete medium and incubated for 24 h with the cells. At the end of the treatment, the medium was removed, and the cells were exposed to 2.5 µg/mL LPS in a complete medium for 4 h at 37 °C and 5% CO_2_. Afterwards, cells were washed twice with PBS and the plate was dried. After drying, the cells were fixed with ice-cold acetone–methanol (1:1) for 10 min. Next, endogenous peroxidases were blocked by adding 100 μL of 0.3% H_2_O_2_ per well for 30 min in the dark. Then, the wells were washed with PBS and blocked by the addition of 1% BSA in PBS for 30 min at 37 °C. After blocking, 50 μL of each primary antibody for IL-1β (source: mouse, 1:500, sc-32294, Santa Cruz Biotechnology, Dallas, TX, USA) and MIF (source: mouse, 1:400, R&D Systems, Birmingham, UK) were added in PBS with 1% BSA to the wells and incubated for 24 h overnight at 4 °C. Following incubation with antibodies, the plate was washed three times with PBS and a secondary antibody (1:2000, Anti-mouse IgG, HRP-linked Antibody 7076S, Cell Signaling Technology, Danvers, MA, USA) in PBS with 1% BSA was added to the wells, and incubation lasted 2 h at room temperature. Finally, the plate was washed three times with PBS, 50 μL of substrate was added to each well, and color development was monitored. When the color developed, 50 μL each of the stop reagent were added and the plate was read at a 450 nm wavelength on a plate reader (BioTek ELx800, Winooski, VT, USA).

### 4.8. Statistical Analysis

In the present study, the analysis of variance (one-way ANOVA) followed by Duncan’s post hoc test (STATISTICA 7.0) were carried out to investigate the statistical significance of the influence of different extraction parameters (ethanol content, solvent volume, and time) on the TPC and differences between values obtained in the assays for determination of total flavonoid, tannin, and protein contents and antioxidant potential. Additionally, according to the results from preliminary screening, the selection of two promising levels of three examined factors, i.e., ethanol concentration, solvent-to-solid ratio, and extraction time (the levels that provided the highest polyphenol yield) was carried out for a further 2^3^ full factorial design to obtain the optimal conditions for each employed extraction procedure for achieving the highest TPC. Each factor was examined at the two most promising levels using the upper and lower limits chosen based on the above-mentioned screening analysis. Specifically, 2^3^ full factorial design (three extraction factors as independent variables at the two most promising levels according to the previous screening) was used to investigate the effect and select the optimal extraction medium (1), solvent-to-solid ratio (2), and period of the extraction process (3) to provide the sample with the highest TPC (a dependent variable). In the factorial design, a 2-level screening design (Box, Hunter, and Hunter) has been conducted to determine the best combination of conditions during the extraction process to prepare the extract with the highest polyphenol concentration. One-way analysis of variance (ANOVA) with the Tukey post hoc test was used to assess differences in treatments versus control after the data were tested for normality in the tests performed on keratinocyte cell lines. All results are expressed as mean ± standard error of the mean (mean ± SEM). GraphPad Prism 6.0 (GraphPad Software, Inc., La Jolla, CA, USA) was used for statistical analysis, where *p* < 0.05 was considered significant.

## 5. Conclusions

In the present study, *F. officinalis* dust extracts with bioactives were developed via optimization of the extraction process and employment of conventional and modern extraction procedures. Moreover, the phytochemical profile and biological effects related to the skin health benefits of the obtained extracts were investigated. Statistical analysis revealed that the type of extraction solvent, solvent-to-solid ratio, and extraction time significantly affected the polyphenol yield of fumitory extracts. Regarding the data of the full factorial design, the optimal conditions to reach the highest level of polyphenol compounds include 50% ethanol and a 30:1 mL/g ratio for 120 s of the extraction in a microwave reactor. The highest total flavonoid, condensed tannin, alkaloid, and protein contents were also measured in the MAE extract. The LC-MS analysis revealed the presence of seven isoquinoline alkaloids, while five of them were characterized as alkaloids of the protopine type (protopine, oxo-, methyl and/or acetyl protopine derivatives, and cryptopine), and two as the spirobenzylisoquinoline type (fumariline and fumarophycine). Chlorogenic and caffeic acids, quercetin trihexoside, quercetin deoxyhexosyl dihexoside, quercetin dihexoside, quercetin pentoside hexoside, rutin, methylquercetin dihexoside, isoquercitrin, methylquercetin pentoside hexoside, kaempferol deoxyhexosylhexoside, methylquercetin deoxyhexosylhexoside, methylquercetin dihexoside, and quercetin were also identified in fumitory dust extracts. Caffeoylmalic acid, quercetin dihexoside, quercetin pentoside hexoside, rutin, and methylquercetin dihexoside were the most dominant compounds. Due to the proven presence of bioactive compounds and the antioxidant and anti-inflammatory activity of fumitory dust extracts, future experiments should include the examination of their other bioactivities (wound-healing, anti-aging, and enzyme-inhibiting effects, etc.) in sophisticated cell-based models of skin diseases, wounds, and aging, with the aim for them to become a promising source for potential dermatological applications. The implementation of fumitory dust extracts into pharmaceutical, cosmetic, and dermo-cosmetic preparations would be possible by proving the aforementioned bioactivities, which would realize the principle of the circular economy—from waste to bioactive formulation or value-added product. Thus, future research can involve the development of various formulations (creams, serums, lotions, masks, etc.), the optimization of the process and composition of formulations, stability studies, *challenge* tests, dermatological testing, and efficiency tests on human volunteers using probe systems to support claims.

## Figures and Tables

**Table 1:** Statistical analysis of optimization of maceration (M) and heat-, ultrasound-, and microwave-assisted extractions (HAE, UAE, and MAE, respectively) from *Fumaria officinalis* using 2^3^ full factorial design (two levels of the following factors: ethanol content, 50 and 70%, solvent-to-solid ratio, 30:1 and 40:1 mL/g, and extraction time, 60 and 90 min of M, 15 and 30 min of HAE, 5 and 15 min of UAE, 120 and 180 s of MAE); the difference was statistically significant at *p* < 0.05.

	Effect	Standard Error	Effect Estimates	Coefficient	Standard Error Coefficient	*p*
M						
TPC * (mg GAE/g)						
Constant				15.270	0.090	0.000
Main factors						
Ethanol content (1)	−2.764	0.180	−15.353	−1.382	0.090	0.000
Solvent-to-solid ratio (2)	−0.247	0.180	−1.375	−0.124	0.090	0.187
Time (3)	−0.062	0.180	−0.347	−0.0312	0.090	0.732
HAE						
TPC (mg GAE/g)						
Constant				18.796	0.086	0.000
Main factors						
Ethanol content (1)	0.307	0.172	1.783	0.154	0.086	0.092
Solvent-to-solid ratio (2)	0.012	0.172	0.072	0.006	0.086	0.943
Time (3)	0.177	0.172	1.029	0.089	0.086	0.318
UAE						
TPC (mg GAE/g)						
Constant				16.911	0.211	0.000
Main factors						
Ethanol content (1)	−3.088	0.422	−7.309	−1.544	0.211	0.000
Solvent-to-solid (2)	−0.893	0.422	−2.114	−0.447	0.211	0.049
Time (3)	0.183	0.422	0.434	0.092	0.211	0.670
MAE						
TPC (mg GAE/g)						
Constant				21.881	0.155	0.000
Main factors						
Ethanol content (1)	−0.405	0.309	−1.310	−0.202	0.155	0.208
Solvent-to-solid ratio (2)	−0.330	0.309	−1.067	−0.165	0.155	0.301
Time (3)	−1.202	0.309	−3.887	−0.601	0.155	0.001

* TPC, total polyphenol content; GAE, gallic acid equivalent.

**Table 2:** Experimental design for screening of extraction parameters for achieving the highest total polyphenol content (TPC), expressed as mg gallic acid equivalent (GAE) per g of dried *Fumaria officinalis* dust in the extracts prepared using maceration (M) and heat-, ultrasound-, and microwave-assisted extractions (HAE, UAE, and MAE, respectively), with measured and predicted values.

Ethanol Content (%)	Solvent-to-Solid Ratio (mL/g)	Time (min or s *)	TPC (mg GAE/g)	
			M	
Parameter levels			Measured value	Predicted value
50	30:1	60	16.56 ± 0.25	16.61 ± 0.50
50	30:1	90	16.92 ± 0.33	16.86 ± 0.50
50	40:1	60	16.56 ± 1.06	16.51 ± 0.50
50	40:1	90	16.58 ± 0.20	16.62 ± 0.50
70	30:1	60	13.98 ± 0.91	14.17 ± 0.50
70	30:1	90	13.87 ± 0.28	13.93 ± 0.50
70	40:1	60	13.86 ± 0.21	13.91 ± 0.50
70	40:1	90	13.58 ± 0.29	13.54 ± 0.50
			HAE	
			Measured value	Predicted value
50	30:1	30	18.33 ± 0.70	18.41 ± 0.48
50	30:1	45	18.72 ± 0.55	18.64 ± 0.48
50	40:1	30	18.72 ± 0.21	18.64 ± 0.48
50	40:1	45	18.80 ± 0.32	18.88 ± 0.48
70	30:1	30	18.19 ± 0.71	18.51 ± 0.48
70	30:1	45	18.30 ± 0.15	18.21 ± 0.48
70	40:1	30	18.51 ± 0.21	18.68 ± 0.48
70	40:1	45	18.39 ± 0.44	18.55 ± 0.48
			UAE	
			Measured value	Predicted value
50	30:1	15	19.04 ± 1.06	19.12 ± 1.18
50	30:1	30	19.59 ± 0.84	19.51 ± 1.18
50	40:1	15	17.44 ± 0.48	17.36 ± 1.18
50	40:1	30	17.76 ± 0.28	17.84 ± 1.18
70	30:1	15	15.54 ± 0.92	15.46 ± 1.18
70	30:1	30	15.27 ± 0.71	15.35 ± 1.18
70	40:1	15	15.26 ± 0.83	15.34 ± 1.18
70	40:1	30	15.40 ± 0.48	15.32 ± 1.18
			MAE	
			Measured value	Predicted value
50	30:1	120	22.95 ± 1.02	22.87 ± 0.86
50	30:1	180	21.39 ± 0.81	21.46 ± 0.86
50	40:1	120	22.56 ± 1.09	22.63 ± 0.86
50	40:1	180	21.43 ± 0.51	21.36 ± 0.86
70	30:1	120	22.41 ± 1.15	22.48 ± 0.86
70	30:1	180	21.47 ± 0.42	21.36 ± 0.86
70	40:1	120	21.55 ± 0.48	21.93 ± 0.86
70	40:1	180	20.86 ± 0.31	20.94 ± 0.86

* Extraction time is expressed in minutes for M, HAE, and UAE, and seconds for MAE.

**Table 3:** The total flavonoid (TFC), condensed tannin (TCTC), and protein contents of *Fumaria officinalis* dust extracts obtained under the optimal extraction conditions in maceration (M) and heat-, ultrasound-, and microwave-assisted extractions (HAE, UAE, and MAE, respectively).

Sample	TFC (mg CE/g)	TCTC (µg CE/g)	Total Proteins (mg AE/g)
M	7.09 ± 0.38 ^c^ *	461.7 ± 18.9 ^c^	5.07 ± 0.30 ^c^
HAE	8.50 ± 0.23 ^b^	715.9 ± 11.8 ^b^	5.39 ± 0.22 ^bc^
UAE	7.51 ± 0.36 ^c^	361.6 ± 15.1 ^d^	5.77 ± 0.35 ^b^
MAE	12.21 ± 0.29 ^a^	755.0 ± 18.3 ^a^	6.50 ± 0.19 ^a^

* Values with the same letter in each column did not significantly differ statistically (one-way analysis of variance and Duncan’s post hoc test, n = 3, *p* > 0.05, mean ± standard deviation). CE, catechin equivalent; AE, albumin equivalent.

**Table 4:** UV and MS spectral data of the detected compounds in *Fumaria officinalis* dust extracts.

No	Rt (min)	λ_max_ (nm)	MV	Paren Ion	Product Ions	Compound
1	9.26–9.38	252, 266sh, 372	788	787	301, 463, 625	Quercetin trihexoside ^(UV,MS)^
2	10.32–10.45	328	354	353	191, 179, 163	Chlorogenic acid ^st^
3	11.11–11.29	252, 266sh, 372	772	771	301, 463, 625	Quercetin deoxyhexosyl dihexoside ^(UV,MS)^
4	15.50–15.67	242, 302, 328	296	295	179, 135, 115	Caffeoylmalic acid ^(UV,MS)^
5	18.43–18.55	256, 268sh, 308sh, 356	626	625	563, 301, 271, 243	Quercetin dihexoside ^(UV,MS)^
6	19.31–19.42	256, 268sh, 308sh, 356	596	595	301, 271, 243	Quercetin pentoside hexoside ^(UV,MS)^
7	20.05–20.17	228, 280, 316	415	416	398, 371, 353, 325, 193, 177, 149	Oxoprotopine derivative [30,31]
8	20.40–20.73	256, 264sh, 354	610	609	301, 271, 243	Quercetin 3-*O*-rutinoside (Rutin) ^st^
9	21.40–21.70	n.d.	640	639	623, 593, 315, 301	Methylquercetin dihexoside ^(UV,MS)^
10	21.50–21.96	256, 264sh, 304sh, 354	464	463	269; 301	Quercetin-3-*O*-glucoside (Isoquercitrin) ^st^
11	22.15–22.20	240, 288, 328	369	370	354, 293, 204, 190	Cryptopine [30,31]
12	22.65–22.70	256, 264sh, 304sh, 354	610	609	315	Methylquercetin pentoside hexoside ^(UV,MS)^
13	22.72–22.80	240, 288, 328	353	354	336, 323, 293, 206, 188, 149, 135	Protopine [30,31]
14	22.67–23.10	266, 348	594	593	285	Kaempferol deoxyhexosylhexoside ^(UV,MS)^
15	23.51–23.63	256, 264sh, 354	624	623	315, 301	Methylquercetin deoxyhexosylhexoside ^(UV,MS)^
16	23.80–23.95	256, 264sh, 354	640	639	315	Methylquercetin dihexoside ^(UV,MS)^
17	24.86–24.90	240, 288, 328	351	352	323, 293, 193	Fumariline [30,31]
18	25.79–25.85	240, 288, 328	337	338	334, 295, 265, 190	Fumarophycine [30,31]
19	28.68–28.80	230, 288, 320	427	428	398, 353, 337, 309, 279, 193, 177	Dimethyl acetyl protopine [30,31]
20	28.92–29.14	n.d.	397	398	382, 353, 335, 325, 295, 206, 193, 165	Acetyl protopine [30,31]
21	29.24–29.36	256, 264sh, 354	302	301	273, 245, 179, 151, 121	Quercetin ^st^

**Table 5:** The content of detected compounds in *Fumaria officinalis* dust extracts.

Compounds	Extracts			
	M	HAE	UAE	MAE
	Content (%, g of compound/100 g of dried extract)			
**1**	<LOQ *	<LOQ	<LOQ	n.d.
**2**	0.095 ± 0.001	0.098 ± 0.005	0.100 ± 0.000	0.078 ± 0.002
**3**	0.119 ± 0.000	0.114 ± 0.008	0.119 ± 0.000	0.126 ± 0.000
**4**	0.537 ± 0.001	0.501 ± 0.004	0.528 ± 0.004	0.465 ± 0.002
**5**	0.395 ± 0.001	0.377 ± 0.003	0.389 ± 0.002	0.403 ± 0.002
**6**	0.723 ± 0.002	0.679 ± 0.001	0.712 ± 0.003	0.712 ± 0.000
**8**	0.717 ± 0.001	0.700 ± 0.006	0.707 ± 0.013	0.752 ± 0.002
**9**	0.532 ± 0.001	0.443 ± 0.004	0.456 ± 0.034	0.398 ± 0.001
**10**	0.197 ± 0.005	0.202 ± 0.001	0.204 ± 0.005	0.214 ± 0.001
**12**	<LOQ	<LOQ	<LOQ	n.d.
**14**	0.100 ± 0.000	0.099 ± 0.009	0.101 ± 0.000	0.071 ± 0.000
**15**	0.034 ± 0.000	0.040 ± 0.004	0.035 ± 0.001	0.015 ± 0.000
**16**	0.089 ± 0.001	0.028 ± 0.001	0.088 ± 0.001	0.088 ± 0.001
**21**	0.088 ± 0.006	0.090 ± 0.000	0.075 ± 0.000	0.104 ± 0.003
Total phenolic fraction	3.631	3.365	3.513	3.426
Total alkaloid fraction	6.885	7.162	9.214	9.322

* The content is calculated as the average of three different determinations ± standard deviations; ˂LOQ—below the limit of quantification. Compound **3** was expressed as caffeic acid; **1**, **3**, **5**, **6**, **9**, **12**, **15**, and **16** as quercetin 3-*O*-rutinoside; **14** as kaempferol 3-*O*-glucoside. Compound names and the most reliable structure are given in Table 4.

## Data Availability

The datasets generated during and/or analyzed during the current study are available from the corresponding author upon reasonable request.

## Supplementary Materials

The following supporting information can be downloaded at: https://www.mdpi.com/article/10.3390/plants14030352/s1. Table S1: Regression equations, correlation coefficients (r^2^), linear ranges, and limits of detection (LOD) and quantification (LOQ) of reference compounds; Figure S1: The effect of ethanol content in the extraction medium, solvent-to-solid ratio, and extraction time on the total polyphenol content (TPC) of *Fumaria officinalis* dust extracts from maceration (M) and heat-, ultrasound-, and microwave-assisted extractions (HAE, UAE, and MAE, respectively), determined by employing one-way analysis of variance followed by the Duncan post hoc test (different letters related to statistically significant difference, *p* < 0.05); Figure S2: Structures of the components identified in *Fumaria officinalis* dust extracts from maceration (M) and heat-, ultrasound-, and microwave-assisted extractions (HAE, UAE, and MAE, respectively) using the LC-MS method—numbers of compounds are presented in Table 4; Figure S3: Chromatograms of selected *Fumaria officinalis* dust extracts prepared using maceration (M) and heat-, ultrasound- and microwave-assisted extractions (HAE, UAE, and MAE, respectively).
