# Supplementary material for: Fumaria officinalis Dust as a Source of Bioactives for Potential Dermal Application: Optimization of Extraction Procedures, Phytochemical Profiling, and Effects Related to Skin Health Benefits
Source: Plants (Basel). 2025 Jan 24;14(3):352. doi: 10.3390/plants14030352 (PMC11819947; doi:10.3390/plants14030352)
Supplement: Supplementary file 1 [file plants-14-00352-s001.zip › plants-3420470-supplementary.pdf]

Table S1. Regression equations, correlation coefficients (r^2), linear ranges, and limits of detection (LOD) and quantification (LOQ) of reference compounds.

Compound	Regression equations	r^2	Linear range (μg/mL)	LOD (μg/mL)	LOQ (μg/mL)
Caffeic acid	y=13866.0695x-2.1700	0.9999	0.07-1.75	0.022	0.066
Chlorogenic acid	y=7359.9706x+21.8506	0.9999	0.05-4.00	0.015	0.044
Quercetin 3-*O*-rutinoside	y=3450.5525x+19.4631	0.9999	0.07-4.00	0.020	0.103
Quercetin 3-*O*-glucoside	y=7039.2891x+22.7091	0.9999	0.62-500	0.035	0.139
Kaempferol 3-*O*-glucoside	y=4007.6421x+5.7835	0.9999	0.16-250	0.031	0.121

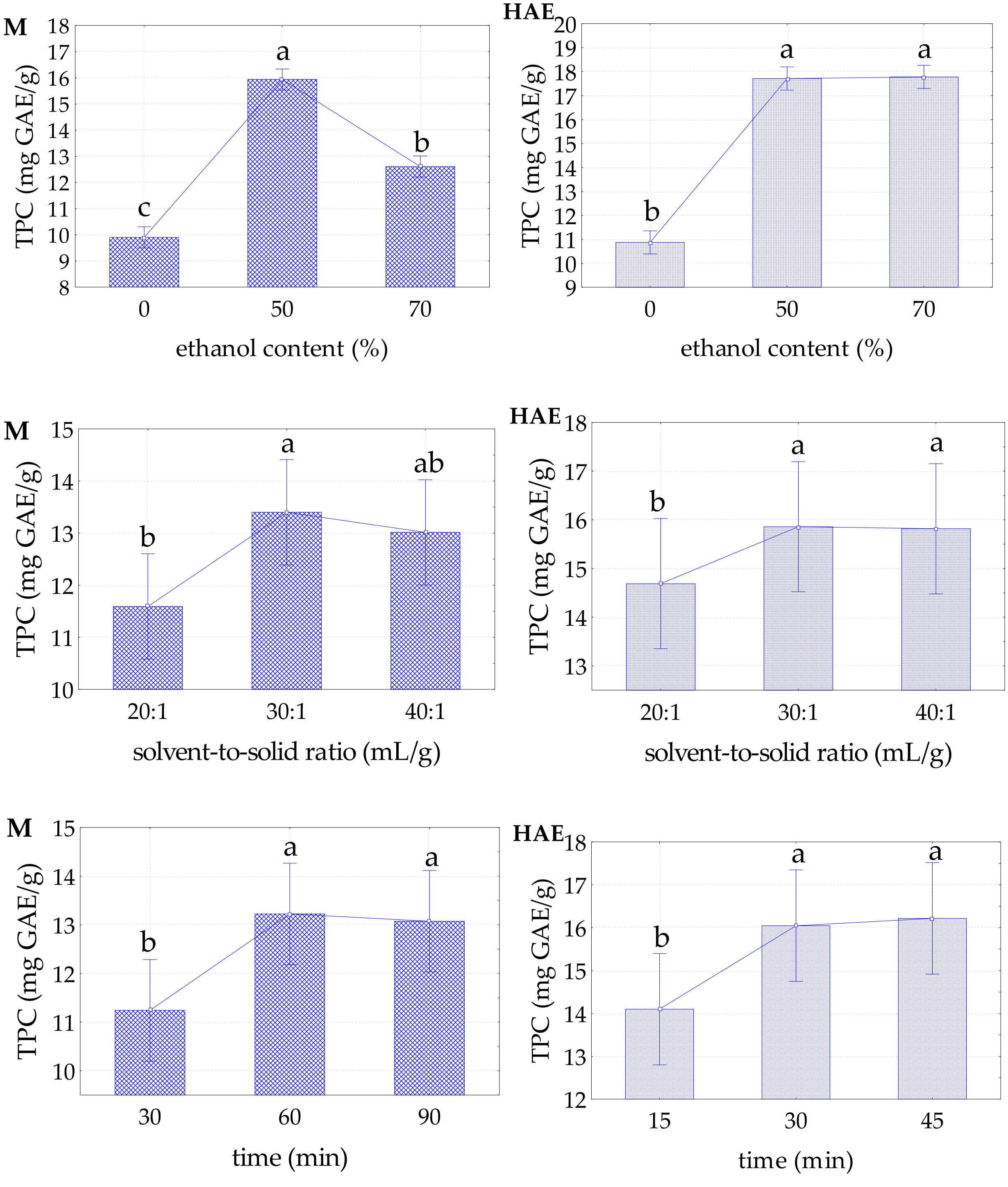

M
TPC (mg GAE/g)
18
17
16
15
14
13
12
11
10
9
8
c
a
b
0
50
70
ethanol content (%)
HAE
TPC (mg GAE/g)
20
19
18
17
16
15
14
13
12
11
10
9
b
a
a
0
50
70
ethanol content (%)
M
TPC (mg GAE/g)
15
14
13
12
11
10
b
a
ab
20:1
30:1
40:1
solvent-to-solid ratio (mL/g)
HAE
TPC (mg GAE/g)
18
17
16
15
14
13
b
a
a
20:1
30:1
40:1
solvent-to-solid ratio (mL/g)
M
TPC (mg GAE/g)
15
14
13
12
11
10
b
a
a
30
60
90
time (min)
HAE
TPC (mg GAE/g)
18
17
16
15
14
13
12
b
a
a
15
30
45
time (min)

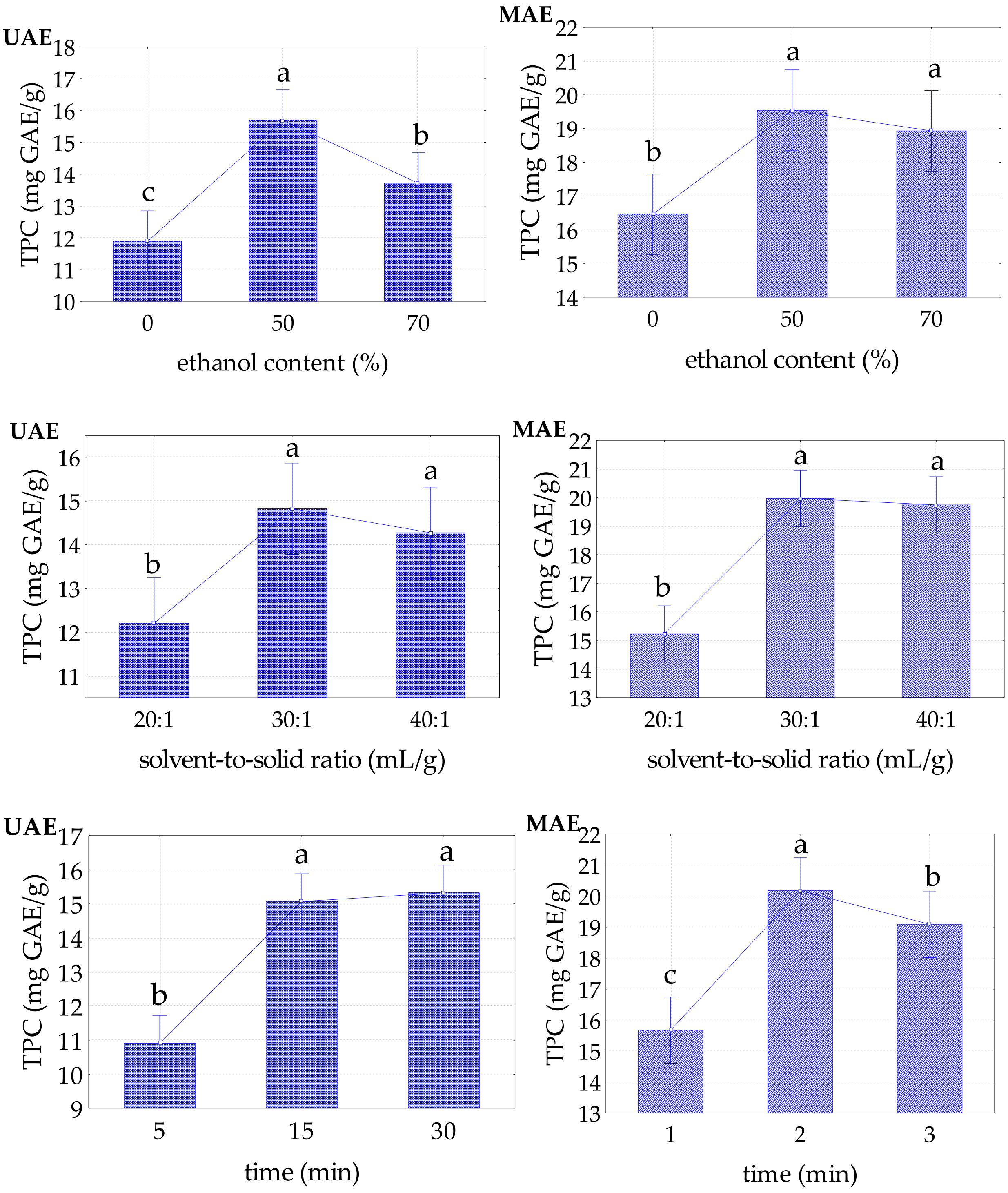

Figure S1. The effect of ethanol content in the extraction medium, solvent-to-solid ratio, and extraction time on the total polyphenol content (TPC) of *Fumaria officinalis* dust extracts from maceration (M) and heat-, ultrasound-, and microwave-assisted extractions (HAE, UAE, and MAE, respectively), determined by employing one-way analysis of variance followed by the Duncan post hoc test (different letters related to statistically significant difference, $p < 0.05$).

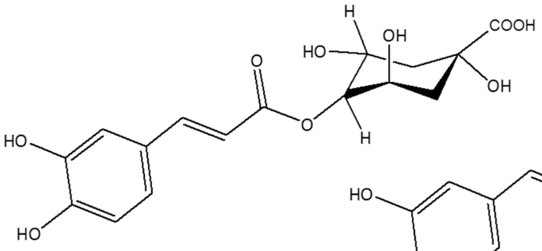

Chlorogenic acid (**2**)

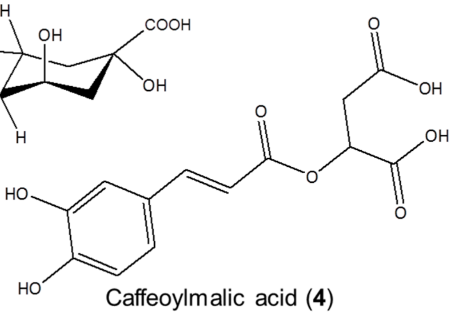

Caffeoylmalic acid (**4**)

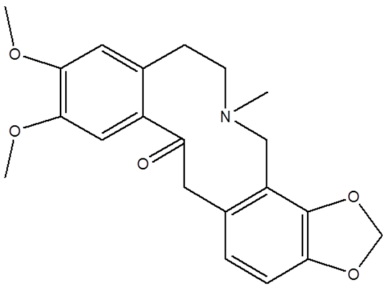

Cryptopine (**11**)

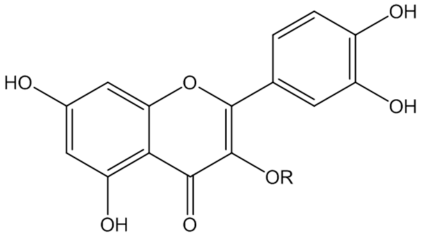

Compound	R
**Rutin (8)**	Rutinose
**Isoquercitrin (10)**	Glucose

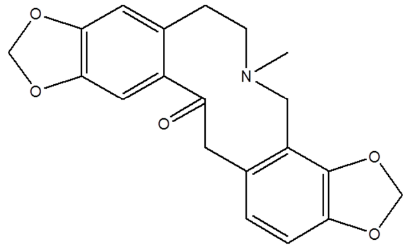

Protopine (**13**)

Figure S2. Structures of the components identified in *Fumaria officinalis* dust extracts from maceration (M) and heat-, ultrasound-, and microwave-assisted extractions (HAE, UAE, and MAE, respectively) using the LC-MS method—numbers of compounds are presented in Table 4.

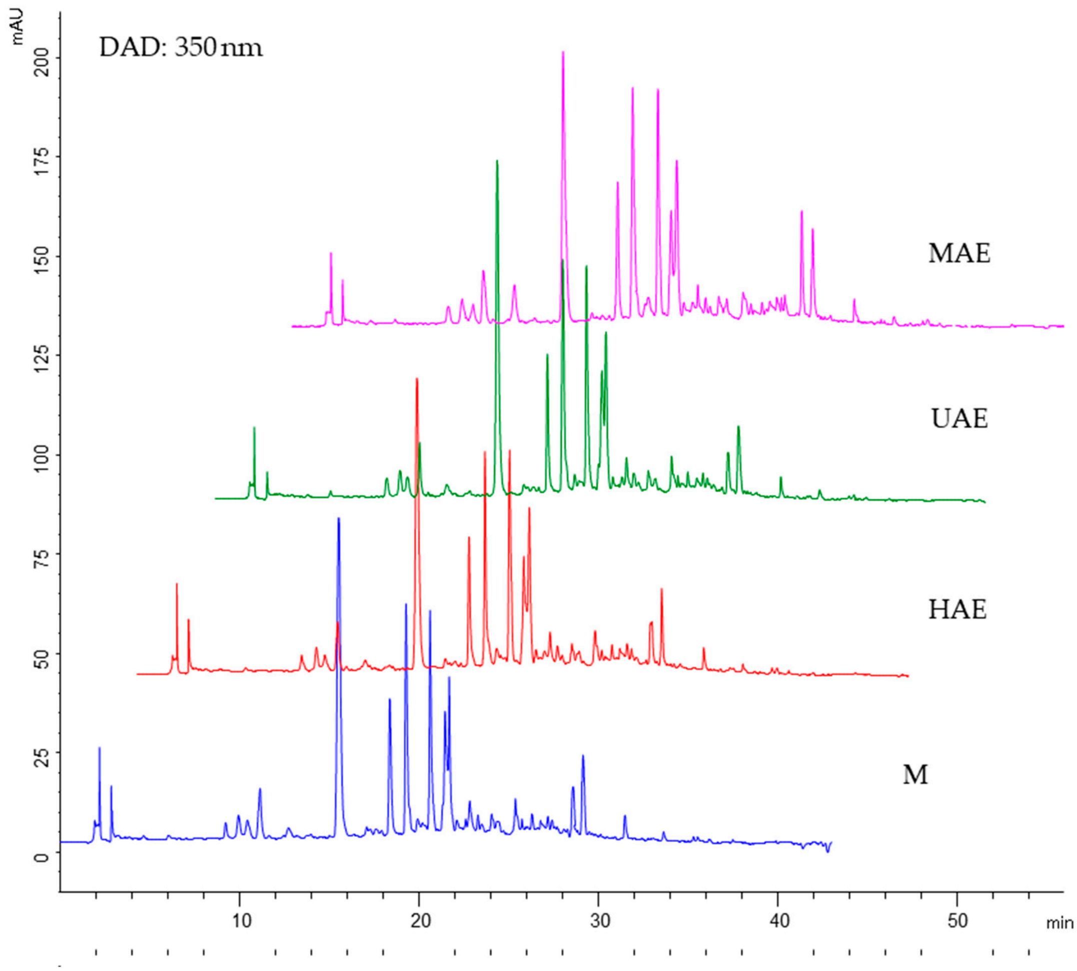

Figure S3. Chromatograms of selected *Fumaria officinalis* dust extracts prepared using maceration (M) and heat-, ultrasound- and microwave-assisted extractions (HAE, UAE, and MAE, respectively).
